# Knock out of specific maternal vitellogenins in zebrafish (*Danio rerio*) evokes vital changes in egg proteomic profiles that resemble the phenotype of poor quality eggs

**DOI:** 10.1186/s12864-021-07606-1

**Published:** 2021-04-28

**Authors:** Ozlem Yilmaz, Amelie Patinote, Emmanuelle Com, Charles Pineau, Julien Bobe

**Affiliations:** 1 INRAE, LPGP, 35000 Rennes, France; 2 Institute of Marine Research, Austevoll Research Station, Storebø, Norway; 3 Univ Rennes, Inserm, EHESP, Irset-UMR_S 1085, F-35042 Rennes cedex, France; 4 Protim, Univ Rennes, F-35042 Rennes cedex, France

**Keywords:** Zebrafish, Vitellogenin, Knock-out, CRISPR/Cas9, Proteomics, LC-MS/MS, Egg quality

## Abstract

**Background:**

We previously reported the results of CRISPR/Cas9 knock-out (KO) of type-I and type-III vitellogenins (Vtgs) in zebrafish, which provided the first experimental evidence on essentiality and disparate functioning of Vtgs at different stages during early development. However, the specific contributions of different types of Vtg to major cellular processes remained to be investigated. The present study employed liquid chromatography and tandem mass spectrometry (LC-MS/MS) to meet this deficit. Proteomic profiles of zebrafish eggs lacking three type-I Vtgs simultaneously (*vtg1*-KO), or lacking only type III Vtg (*vtg3*-KO) were compared to those of wild type (Wt) eggs. Obtained spectra were searched against a zebrafish proteome database and identified proteins were quantified based on normalized spectral counts.

**Results:**

The *vtg*-KO caused severe changes in the proteome of 1-cell stage zebrafish eggs. These changes were disclosed by molecular signatures that highly resembled the proteomic phenotype of poor quality zebrafish eggs reported in our prior studies. Proteomic profiles of *vtg*-KO eggs and perturbations in abundances of hundreds of proteins revealed unique, noncompensable contributions of multiple Vtgs to protein and in energy homeostasis. The lack of this contribution appears to have a significant impact on endoplasmic reticulum and mitochondrial functions, and thus embryonic development, even after zygotic genome activation. Increased endoplasmic reticulum stress, Redox/Detox activities, glycolysis/gluconeogenesis, enrichment in cellular proliferation and in human neurodegenerative disease related activities in both *vtg1*- and *vtg3*-KO eggs were found to be indicators of the aforementioned conditions. Distinctive increase in apoptosis and Parkinson disease pathways, as well as the decrease in lipid metabolism related activities in *vtg3*-KO eggs implies compelling roles of Vtg3, the least abundant form of Vtgs in vertebrate eggs, in mitochondrial activities. Several differentially abundant proteins representing the altered molecular mechanisms have been identified as strong candidate markers for studying the details of these mechanisms during early embryonic development in zebrafish and possibly other vertebrates.

**Conclusions:**

These findings indicate that the global egg proteome is subject to extensive modification depending on the presence or absence of specific Vtgs and that these modifications can have a major impact on developmental competence.

**Supplementary Information:**

The online version contains supplementary material available at 10.1186/s12864-021-07606-1.

## Background

Multiple forms of vitellogenins (Vtg) constitute the major source of egg yolk nutrients supporting development of oviparous animals. The multiple Vtg system in spiny-rayed teleosts (Acanthomorpha) consists of two paralogous complete forms of VtgA (VtgAa, and VtgAb) as well as an incomplete form of Vtg, VtgC [1–3]. Based on prior reports [4–7], the complex repertoire of multiple *vtg* genes in zebrafish includes five type-I *vtgs* (*vtgs1, 4, 5, 6,* and *7*), two type-II *vtgs* (*vtg2, and vtg8*), and one type-III *vtg* (*vtg3*). The different major forms of zebrafish Vtg protein (type I, II and III) are orthologs of the multiple Vtgs present in acanthomorphs (VtgAa, VtgAb and VtgC, respectively).

Vtgs may be complete or incomplete with respect to the presence or absence of certain yolk protein (YP) domains in their primary structure. Complete forms of teleost Vtgs consist of the following yolk protein domains; NH_2_, lipovitellin heavy chain (LvH), phosvitin (Pv), lipovitellin light chain (LvL), β’-component (β’-c), carboxy-terminal component (Ct), COOH [8]. There is very limited knowledge on the specific functions of the different YPs. Lipovitellin, a large lipoprotein carrying primarily structural phospholipids, is the major Vtg-derived YP. Phosvitin, a small YP made up mostly of phosphorylated serine residues, is thought to stabilize the Lv structure, enhance lipid loading of Lv, promote Vtg solubility, and act as a carrier of divalent cations (e.g. Ca^2+^) into the oocytes [9]. The two diminutive domains, β’-c and Ct, possess a highly conserved array of disulfide bonds that are thought to be important for stabilizing Vtg structure, promoting Vtg dimerization, and enabling Vtg receptor (VtgR) binding. They may persist or be degraded during oocyte maturation. However, their nutritional function is still unknown. Incomplete forms of Vtg, such as the Vtg3 in zebrafish, possess both Lv domains (LvH + LvL) but may lack any or all of the remaining YP domains.

The roles that different types of Vtg play in oocyte growth and maturation, and in embryonic and larval development, has been a target of attention for decades [10–12]. The Vtgs are preferentially produced by the liver and transported via the bloodstream to the ovary into which they are taken up via receptor-mediated endocytosis during the process termed vitellogenesis [13–15]. Here they are processed into product yolk proteins by the lysosomal endopeptidase, cathepsin D (CatD), and are stored in the ooplasm during oocyte growth [16–18].

A second selective proteolysis of yolk protein products into free amino acids (FAA) by other cathepsins (e.g. CatB, L) takes place during oocyte maturation. The resulting increase in FAA plays a significant role as an osmotic effector inducing water influx into oocytes of marine species spawning pelagic eggs [2, 15, 19–22]. Moreover, the FAA are critical nutrients, serving as substrates for energy production and for protein synthesis during early embryogenesis [23–29].

The degree of maturational yolk proteolysis, and the YP products of different types of Vtg that are subjected to proteolysis, differ among species with eggs having disparate physiological properties leading to different environmental adaptations (i.e. demersal versus pelagic eggs). In barfin flounder, (*Verasper moserii*), all YPs derived from VtgAa are completely degraded into FAA while those derived from the corresponding VtgAb and VtgC are less susceptible to proteolysis [10, 30]. The relatively intact major VtgAb and VtgC polypeptides (LvHs) escaping proteolysis are ascribed to be specialized for delivering large lipoprotein nutrients to late stage larvae without affecting the osmotically active FAA pool [3, 11]. However, studies of temperate basses (family *Moronidae*) have revealed that, in other acanthomorphs, maturational yolk proteolysis may instead involve limited degradation of all three forms of LvH (LvHAa, LvHAb, LvHC) [3, 11, 31, 32]. Furthermore, results of our recent study of zebrafish examining the effects of simultaneous knock-out of multiple type I *vtgs*, versus the effects of KO of *vtg3* only, revealed substantial mortality of *vtg3*-KO eggs/embryos after only 8 h post-fertilization, whereas significant mortality of *vtg1*-KO embryos did not occur until ~ 5 days later. These observations and similar disparities seen during late larval stages provided the first experimental evidence that different types of Vtg are essential and have disparate requisite functions at different times of development in zebrafish, a well-established biomedical model for research on reproduction and developmental biology [33].

Despite the high interest in the subfunctionalization of multiple forms of Vtgs among fishes, there remains a general lack of knowledge on specific contributions of different types of Vtg to disparate molecular functions related to major developmental processes. Therefore, the main objectives of this study were to evaluate any differences in the proteomic profiles of eggs obtained from zebrafish with and without specific types of Vtgs, including wild type fish (Wt), *vtg1*-KO fish lacking *vtg1*, *4*, *5* in their genome, and *vtg3*-KO fish lacking *vtg3* in their genome. With these comparisons we aimed to reveal whether the absence of specific types of Vtg may influence the global proteome of the newly fertilized egg, and thereby alter requisite molecular mechanisms of early embryonic development.

## Results

### Frequency distribution of differentially regulated proteins

**Fig. 1:**
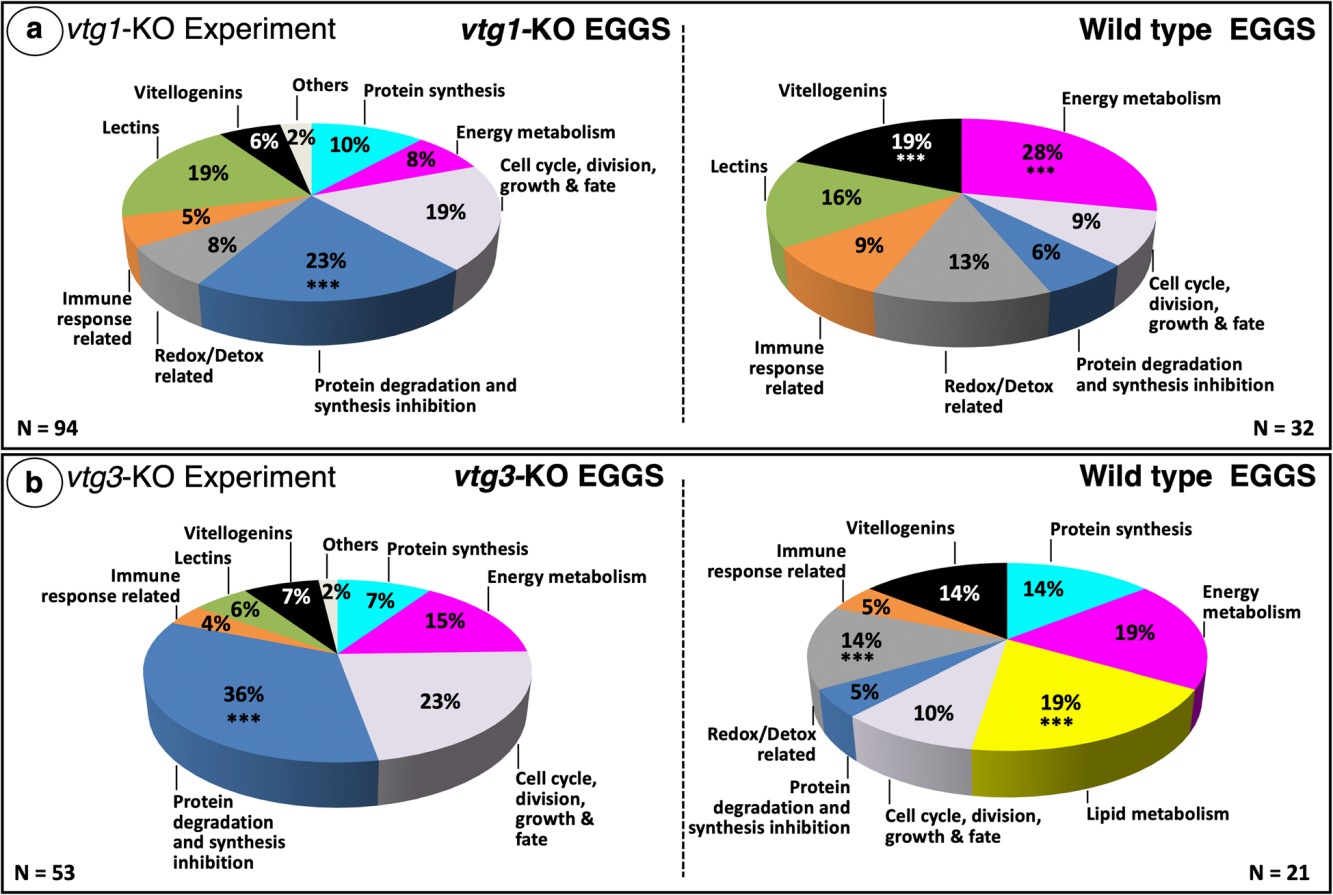
Distribution of differentially regulated proteins among functional categories. Panel a. *vtg1*-KO Experiment. Panel b. *vtg3*-KO Experiment. Only proteins that were identified in > 4 biological samples and that exhibited a > 1.5-fold difference in N-SC between groups (*vtg*-KO versus Wt), or proteins unique to a certain group, were included in this analysis. In both experiments, the overall distribution of differentially regulated proteins among the functional categories significantly differed between KO and Wt eggs (χ^2^, *p* < 0.05). Asterisks indicate significant differences between different groups in the proportion of differentially regulated proteins within a functional category (χ^2^, *p* < 0.05). The corresponding Ensembl Protein IDs and associated gene, transcript and protein names, functional categories (shown above), regulation (compared to Wt) (unique, up-regulated in KO or down-regulated in KO), and fold-difference in N-SC between KO and Wt eggs for proteins included in this analysis for the *vtg1*-KO and *vtg3*-KO experiments are given in Tables S1 and S2, respectively

When extracts of newly fertilized eggs from *N* = 4 F3 homozygous *vtg1*-KO females and from *N =* 4 Wt females were compared in terms of their proteomic profiles via label-free LC-MS/MS, a total of *N* = 301 proteins were identified. Of these, *N* = 126 proteins were detected in at least four samples and showed a ≥ 1.5-fold difference in normalized spectral counts (N-SC) between groups (*vtg1*-KO versus Wt), or were unique to a certain group, and, on this basis, were considered to be ‘differentially regulated’ (Table S1). Accordingly, proteins which were detected with lower abundance in *vtg*-KO eggs in comparison to Wt eggs were reported as “down-regulated in KO”, and those which were detected with higher abundance in *vtg*-KO eggs in comparison to Wt eggs were reported as “up-regulated in KO” (Tables S1 and S2). The frequency distribution of differentially regulated proteins among 10 categories of physiological function chosen to represent most (> 90%) of the proteins significantly differed (χ^2^, *p* < 0.05) between *vtg1*-KO and Wt eggs (Fig. 1a). Frequencies of proteins related to protein degradation and synthesis inhibition were significantly higher, whereas frequencies of proteins related to energy metabolism and vitellogenins were significantly lower in *vtg1*-KO eggs in comparison to Wt eggs. Individual proteins that were up-regulated in *vtg1*-KO eggs (*vtg1*-KO; *N* = 94) (Fig. 1a) were mainly related to protein degradation and synthesis inhibition (23%), cell cycle, division, growth and fate (19%), lectins (19%), and protein synthesis (10%), with the remaining categorized proteins being related to energy metabolism (8%), redox-detox activities (8%), vitellogenins (6%) and immune functions (5%). Two percent of proteins which were up-regulated in *vtg1*-KO eggs were placed in the category of ‘others’. Individual proteins that were down-regulated in *vtg1*-KO eggs (Wt; *N* = 32) were mainly related to energy metabolism (28%), Vtgs (19%), lectins (16%), and redox-detox activities (13%) with the remaining categorized proteins being related to cell cycle, division, growth and fate (9%), immune functions (9%), and protein degradation and synthesis inhibition (6%).

When protein extracts of eggs from *N* = 4 F3 homozygous *vtg3*-KO females, and from *N =* 4 Wt females, were compared in terms of their protein profiles, a total of *N* = 238 proteins were identified. Based on the criteria mentioned above, *N* = 74 of these proteins were identified as ‘differentially regulated’ (Table S2). The frequency distribution of differentially regulated proteins among the 10 categories of physiological function chosen to represent most (> 90%) of the proteins significantly differed (χ^2^, *p* < 0.05) between *vtg3*-KO and Wt eggs (Fig. 1b). The frequency of proteins related to protein degradation and synthesis inhibition was significantly higher, whereas the frequencies of proteins related to lipid metabolism, and to redox-detox activities were significantly lower in *vtg3*-KO eggs in comparison to Wt eggs (χ^2^, *p* < 0.05). Individual proteins that were up-regulated in *vtg3*-KO eggs (*vtg3*-KO; *N* = 53) were mainly related to protein degradation and synthesis inhibition (36%), cell cycle, division, growth and fate (23%), and energy metabolism (15%) with the remaining categorized proteins being related to protein synthesis (7%), Vtgs (7%), lectins (6%), and immune functions (4%). Two percent of these proteins were placed in the ‘others’ category. Individual proteins that were down-regulated in *vtg3*-KO eggs (Wt; *N* = 21) were mainly related to lipid metabolism (19%), energy metabolism (19%), protein synthesis (14%), redox-detox activities (14%) and vitellogenins (14%), with the remaining proteins being related to immune function (5%) and protein degradation and synthesis inhibition (5%).

### PANTHER GO overrepresentation analysis of differentially regulated proteins

**Fig. 2:**
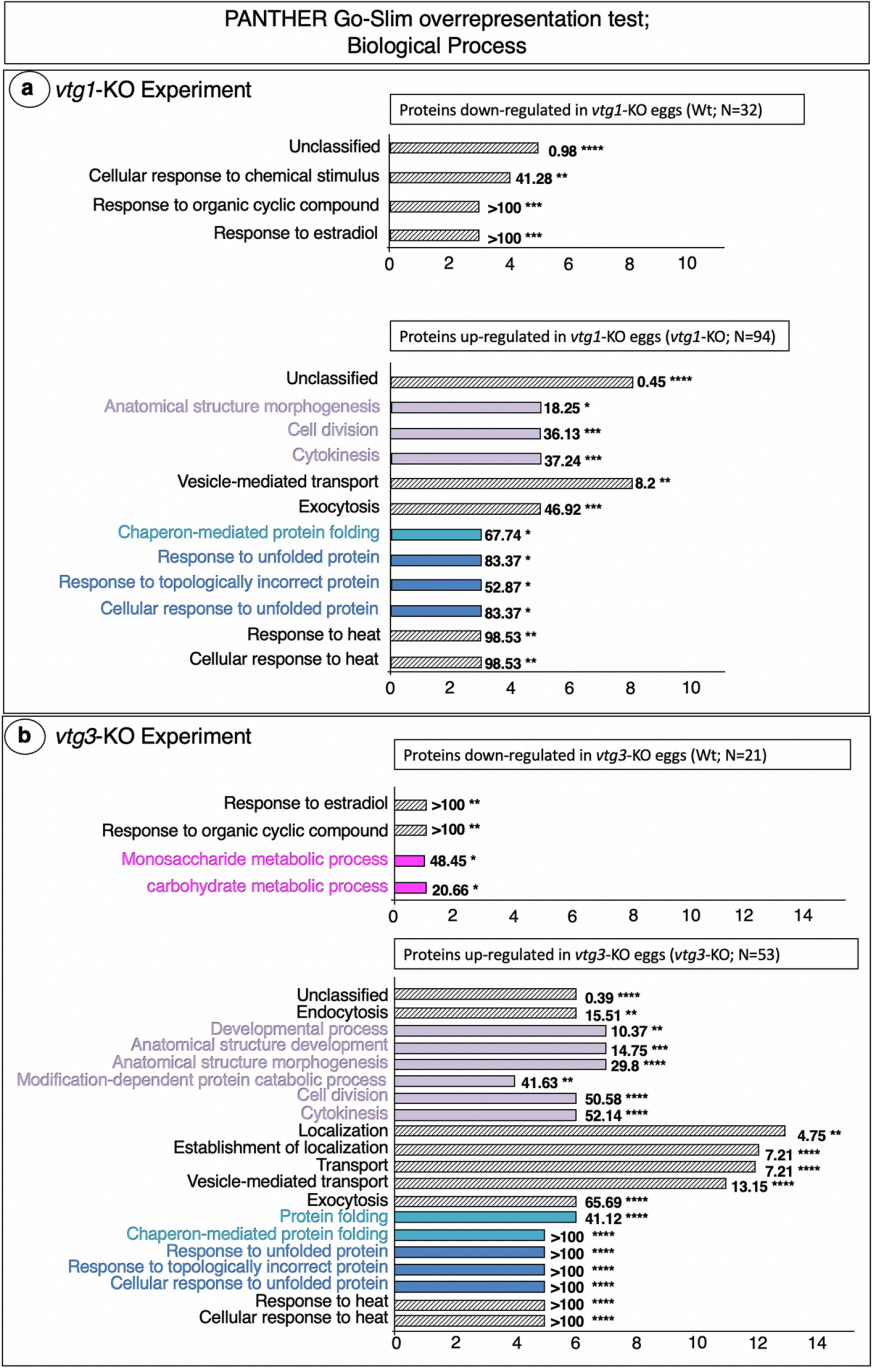
PANTHER GO Biological Processes found to be overrepresented by differentially regulated proteins. Panel a. *vtg1*-KO Experiment. Panel b. *vtg3*-KO Experiment. Only proteins that were identified in > 4 biological samples and that exhibited a > 1.5-fold difference in N-SC between groups (*vtg1*-KO versus Wt), or proteins unique to a certain group, were included in this analysis. Horizontal bars indicate the number of proteins attributed to each GO term for which statistically significant results (Fisher’s Exact test, *p* < 0.05, followed by Bonferroni correction for multiple testing (*p <* 0.05)) were observed (Results shown for Wt egg proteins in Fig. 2b. Top Panel based on FDR only, no Bonferroni correction was applied). Numbers next to the bars indicate the fold-enrichment with proteins attributed to each term and the number of asterisks indicates the significance level of the enrichment, as follows *p* < 0.05 (*), *p* < 0.01 (**), *p* < 0.001 (***), and *p* < 0.0001 (****). Where possible, horizontal bars are colored to indicate corresponding protein functional categories shown in Fig. 1; cell cycle, division, growth and fate (lavender), protein synthesis (light blue), protein degradation and synthesis inhibition (dark blue), energy metabolism (magenta)

**Fig. 3:**
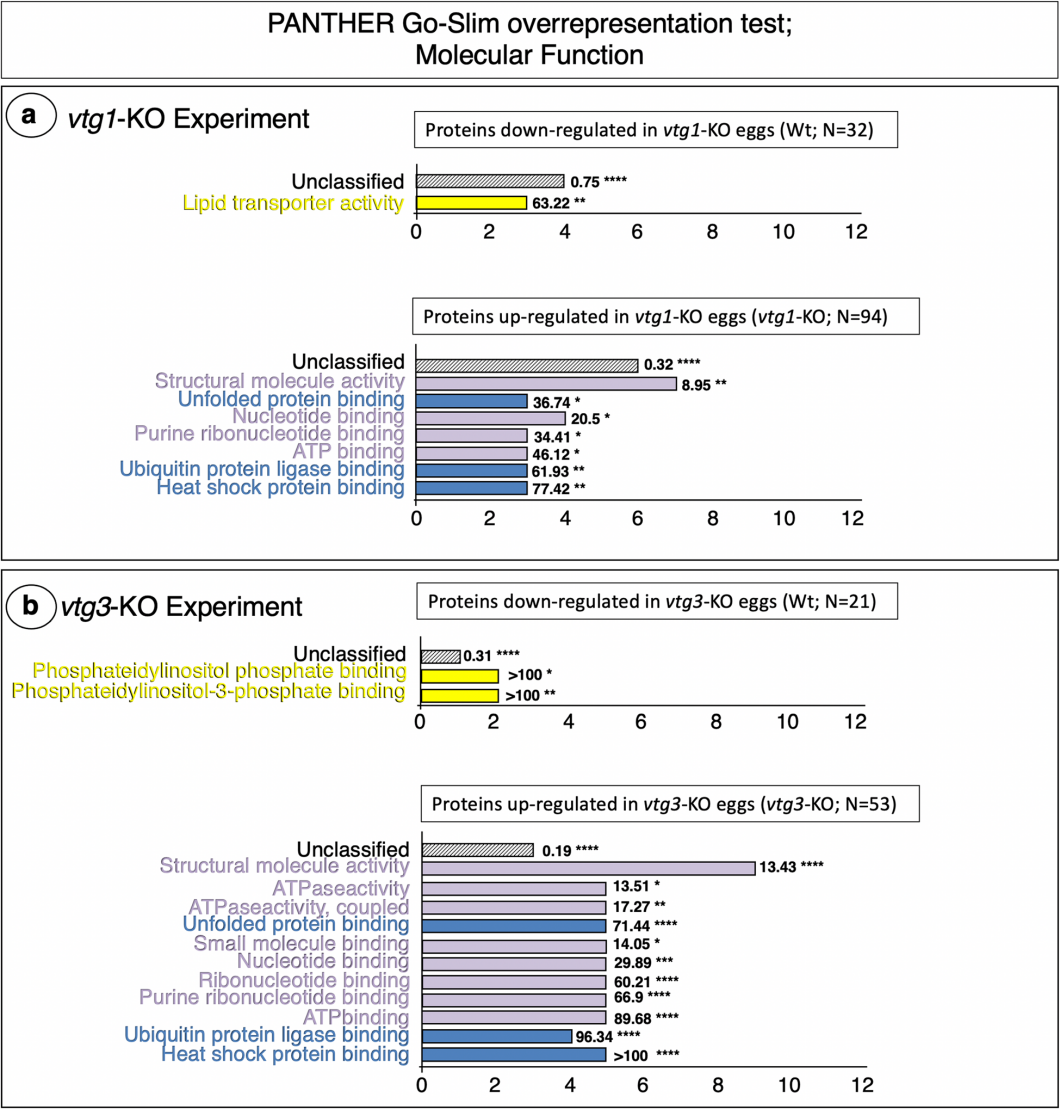
PANTHER GO Molecular Functions found to be overrepresented by differentially regulated proteins. Panel a. *vtg1*-KO Experiment. Panel b. *vtg3*-KO Experiment. Only proteins that were identified in > 4 biological samples and that exhibited a > 1.5-fold difference in N-SC between groups (*vtg3*-KO versus Wt), or proteins unique to a certain group, were included in this analysis. Horizontal bars indicate the number of proteins attributed to each GO term for which statistically significant results (Fisher’s Exact test, *p* < 0.05, followed by Bonferroni correction for multiple testing (*p <* 0.05)) were observed. Numbers next to the bars indicate the fold-enrichment with proteins attributed to each term and the number of asterisks indicates the significance level of the enrichment, as follows *p* < 0.05 (*), *p* < 0.01 (**), *p* < 0.001 (***), and *p* < 0.0001 (****). Where possible, horizontal bars are colored to indicate corresponding protein functional categories shown in Fig. 1; lipid metabolism (yellow), cell cycle, division, growth and fate (lavender), protein degradation and synthesis inhibition (dark blue)

An automated *Protein ANalysis THrough Evolutionary Relationships* (PANTHER) overrepresentation test of the 126 proteins regulated differentially between *vtg1*-KO and Wt eggs revealed three Gene Ontology (GO) Biological Process terms, Cellular response to chemical stimulus, Response to organic cyclic compound and Response to estradiol, to be significantly overrepresented by proteins down-regulated in *vtg1*-KO eggs (Fig. 2a. Top Panel). The GO Molecular Function term, Lipid transporter activity, was also significantly overrepresented by proteins down-regulated in *vtg1*-KO eggs (Fig. 3a. Top Panel). In contrast, many Biological Process terms were significantly overrepresented by proteins up-regulated in *vtg1*-KO eggs. These included mainly terms related to cell cycle, division, growth and fate (Anatomical structure morphogenesis, Cell division, Cytokinesis), to protein synthesis (Chaperone-mediated protein folding), and to protein degradation and synthesis inhibition (Response to unfolded protein, Response to topologically incorrect protein, Cellular response to unfolded protein, Response to heat, Cellular response to heat) (Fig. 2a. Bottom Panel). The remaining terms were related to vesicle-mediated transport (Vesicle-mediated transport, Exocytosis). Similarly, the several GO Molecular Function terms that were significantly overrepresented by proteins up-regulated in *vtg1*-KO eggs were related to cell cycle, division, growth and fate (Structural molecule activity, Nucleotide binding, Purine ribonucleotide binding, ATP binding), or to protein degradation and synthesis inhibition (Unfolded protein binding, Ubiquitin protein ligase binding, Heat shock protein binding) (Fig. 3a. Bottom Panel). The broad agreement between Biological Process and Molecular Function terms is consistent with a proteome tailored to cytoskeletal activities and to protein degradation and synthesis inhibition shown in Fig. 1a. Accordingly, a PANTHER analyses conducted to identify GO Cellular Component and Protein Class terms overrepresented by proteins up-regulated in *vtg1*-KO eggs returned mainly proteins related to cytoskeletal regulation and protein degradation and synthesis inhibition (Table S3).

A PANTHER overrepresentation test of the 74 proteins differentially regulated between *vtg3*-KO and Wt eggs revealed the GO Biological Process terms, Response to estradiol, Response to organic cyclic compound, Monosaccharide metabolic process and Carbohydrate metabolic processes to be overrepresented by proteins down-regulated in *vtg3*-KO eggs (Fig. 2b. Top Panel). The Molecular Function terms, Phosphatidylinositol phosphate binding and Phosphatidylinositol-3-phosphate binding were significantly over represented by proteins down-regulated in *vtg3*-KO eggs (Fig. 3b. Top Panel), which is consistent with the proteomic emphasis on lipid metabolism activities revealed by the frequency distribution analysis (Fig. 1b), and also with the overrepresentation of the Molecular Function term, Lipid transporter activity, by proteins down-regulated in KO eggs in the *vtg1*-KO experiment (Fig. 3a. Top Panel). Similar to the results of the *vtg1*-KO experiment, many GO Biological Process terms were significantly overrepresented by proteins up-regulated in *vtg3*-KO eggs, and these included mainly terms related to Cell cycle, division, growth and fate (Developmental process, Anatomical structure development, Anatomical structure morphogenesis, Modification-dependent protein catabolic process, Cell division, cytokinesis), to protein synthesis (Protein folding, Chaperone-mediated protein folding), and to Protein degradation and synthesis inhibition (Response to unfolded protein, Response to topologically incorrect protein, Cellular response to unfolded protein, Response to heat, Cellular response to heat), as well as some terms related to vesicle trafficking (Endocytosis, Transport, Vesicle-mediated transport, Exocytosis) (Fig. 2b. Bottom Panel). As was the case with proteins up-regulated in *vtg1*-KO eggs, all of the many Molecular Function terms that were significantly overrepresented by proteins up-regulated in *vtg3*-KO eggs were related to cell cycle, division, growth and fate (Structural molecule activity, ATPase activity, ATPase activity-coupled, Small molecule binding, Nucleotide binding, Ribonucleotide binding, Purine ribonucleotide binding, ATP binding), or to Protein degradation and synthesis inhibition (Unfolded protein binding, Ubiquitin protein ligase binding, Heat shock protein binding) (Fig. 3b. Bottom Panel). A PANTHER analysis conducted to identify GO Cellular Component and Protein Class terms overrepresented by proteins down-regulated in *vtg3*-KO eggs returned only the membrane components Autophagosome and Lysosomal membrane, whereas the same analysis for proteins up-regulated in *vtg3*-KO eggs returned Actin cytoskeleton component, many cytoskeletal protein classes and class Ribosomal protein (Table S4).

**Fig. 4:**
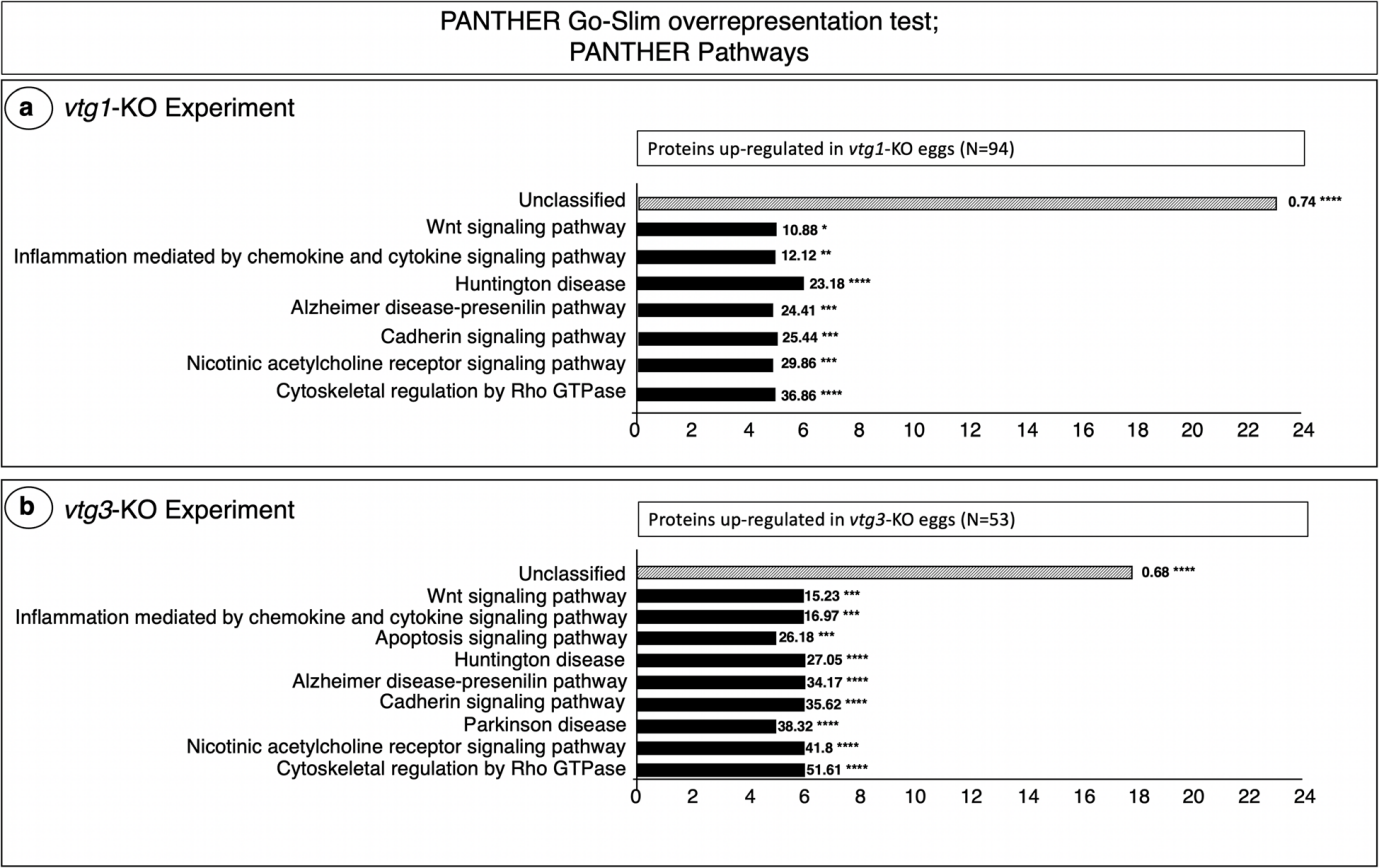
PANTHER GO Biological Pathways which are significantly over-represented by proteins up-regulated in *vtg*-KO eggs Panel a. *vtg1*-KO Experiment Panel b. *vtg3*-KO Experiment. Only proteins which were identified in > 4 biological samples and those with a > 1.5-fold difference in N-SC, or proteins unique to any group, were included in this analysis. Horizontal bars indicate the number of proteins attributed to each pathway for which statistically significant results (Fisher’s Exact test, *p* *<* 0.05, followed by Bonferroni correction for multiple testing (*p <* 0.05)) were observed. Numbers next to the bars indicate the fold-enrichment with proteins attributed to each pathway and the number of asterisks indicates the significance level of the enrichment, as follows *p* < 0.05 (*), *p* < 0.01 (**), *p* < 0.001 (***), and *p* < 0.0001 (****)

A PANTHER Pathways overrepresentation analysis revealed the Wnt signaling, Inflammation mediated by chemokine and cytokine signaling, Huntington disease, Alzheimer disease-presenilin, Cadherin signaling, Nicotinic acetylcholine receptor signaling, and Cytoskeletal regulation by Rho GTPase pathways to be significantly overrepresented by proteins up-regulated in *vtg1*-KO eggs (Fig. 4a). All of these pathways were also significantly overrepresented by proteins up-regulated in *vtg3*-KO eggs (Fig. 4b). The Apoptosis signaling pathway, and the Parkinson disease pathway, were also overrepresented by proteins up-regulated in *vtg3*-KO eggs. The PANTHER Pathways analyses revealed no pathways to be significantly overrepresented by proteins with higher abundance in Wt eggs and so down-regulated in KO eggs for both experiments.

### STRING functional protein association networks analysis of differentially regulated proteins

**Fig. 5:**
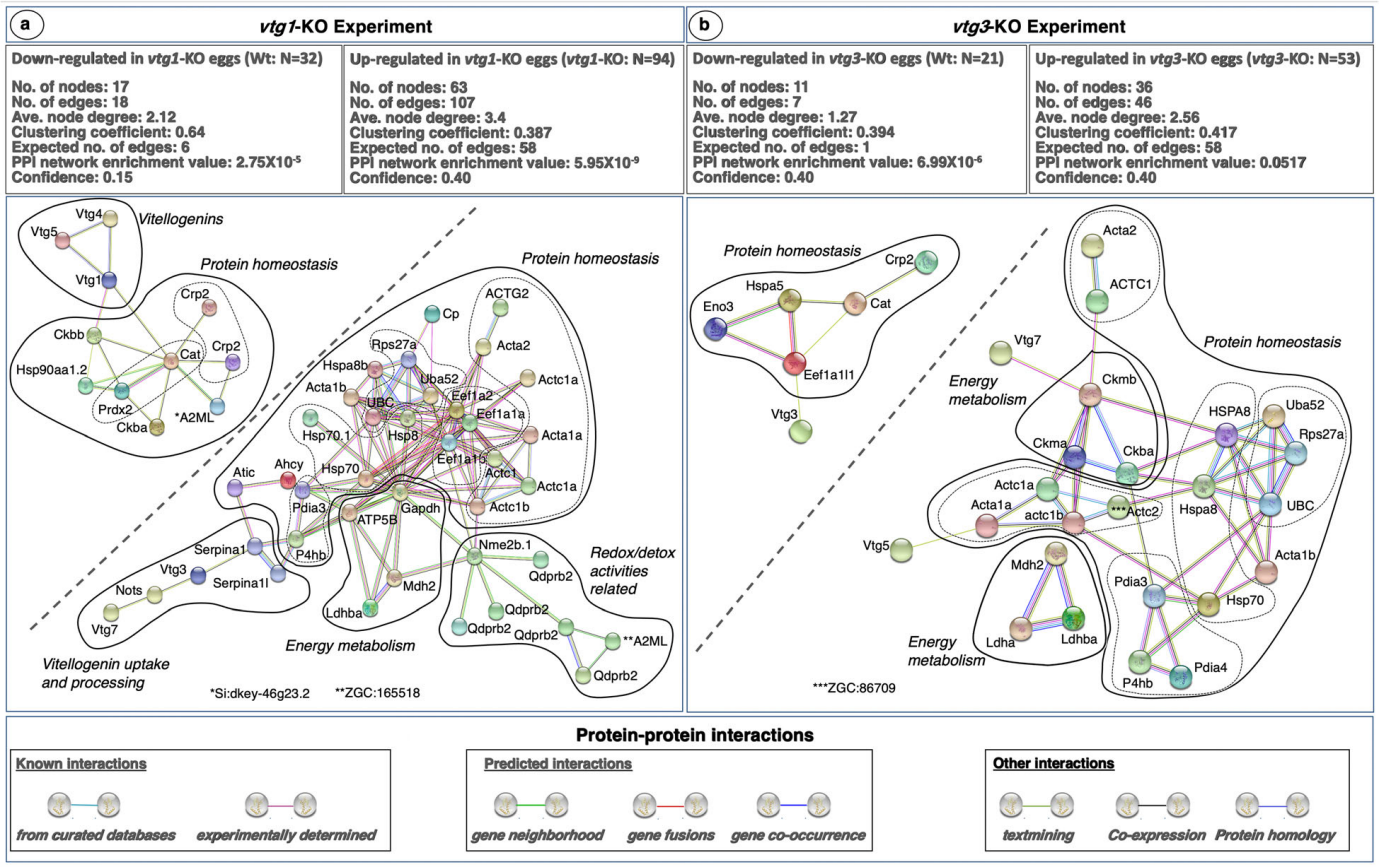
STRING Network Analysis of the differentially regulated proteins in *vtg1*-KO and *vtg3*-KO experiments. A total of 32 proteins which were down-regulated in *vtg1*-KO eggs and 94 proteins which were up-regulated in *vtg1*-KO eggs in *vtg1*-KO experiment, and a total of 21 proteins which were down-regulated in *vtg3*-KO eggs and 53 proteins which were up-regulated in *vtg3*-KO eggs in *vtg3*-KO experiment, were over-represented in specific biological pathways (Tables 1 and 2). Each network node (sphere) represents all proteins produced by a single, protein-coding gene locus (splice isoforms or post-translational modifications collapsed). Only nodes representing query proteins are shown. Nodes are named for the transcript(s) to which spectra were mapped; for full protein names, see Tables S1 and S2. Edges (colored lines) represent protein-protein associations meant to be specific and meaningful, e.g. proteins jointly contribute to a shared function but do not necessarily physically interact. Model statistics are presented at the top left and at the top right of each panel for proteins down- and up-regulated in KO eggs, respectively. Explanation of edge colors is given below panels. For each experiment, the subnetwork formed by proteins down-regulated in KO eggs is shown to the upper left above the diagonal dashed line, and the subnetwork formed by proteins up-regulated in KO eggs is shown to the lower right below the diagonal dashed line. Where possible, solid lines encircle clusters of transcripts encoding interacting proteins involved in physiological processes distinct from other such clusters (see text for details). Dashed lines identify subclusters of two or more transcripts encoding proteins of a common type

When the 126 differentially regulated proteins with significant differences in abundance between *vtg1*-KO and Wt eggs were submitted separately (Wt; *N* = 32, *vtg1*-KO; *N* = 94) to a functional protein association networks analysis using the *Search Tool for the Retrieval of Interacting Genes/Proteins* (STRING) and the zebrafish protein database, they resolved into networks with significantly and substantially greater numbers of known and predicted interactions between proteins than would be expected of the same size lists of proteins randomly chosen from the zebrafish database (Fig. 5a). The subnetwork formed by proteins down-regulated in *vtg1*-KO eggs (Wt; *N *= 32) is made up of, a cluster of three type-I Vtgs (Vtg1, Vtg4 and Vtg5) loosely interacting with a second cluster having at its center two strongly interacting peroxidases, catalase (Cat) and a peroxiredoxin (Prdx2), responsible for protecting the cell from oxidative damage by reactive oxygen species (ROS). Catalase interacts with all remaining members of the cluster, including two C-reactive proteins (Crp2), pattern recognition receptors that activate the complement system, a broad spectrum antiprotease, alpha-2-macroglobulin-like (A2ML), two forms of creatinine kinase (Ckba, Ckbb), an enzyme essential for cellular energy homeostasis, and the common heat shock protein (Hsp90), a key regulator of proteostasis including protein folding, stabilization and degradation when damaged. Aside from Cat, the Prdx2 reacted only with Hsp90 and the two creatinine kinases (Fig. 5a. Left Panel, PPI network enrichment value *P* = 2.75 × 10^− 5^).

The subnetwork formed by proteins up-regulated in *vtg1*-KO eggs (*vtg1*-KO; *N* = 94) is made up of four interacting protein clusters. The first cluster includes two vitellogenins (Vtg7 and Vtg3), an aspartic protease, nothepsin (Nots), secreted by the liver of vitellogenic females and stored in the egg yolk, and two serpins (Serpina1 and Serpina 1I), broad spectrum serine protease inhibitors produced by the liver of vitellogenic females, that bind to Vtgs for transport into the yolk where they associate with the surface of Vtg-derived yolk platelets. The second cluster includes several proteins related to energy metabolism, including glyceraldehyde-3-phosphate dehydrogenase (Gapdh), lactate dehydrogenase (Ldhba), malate dehydrogenase (Mdh2), and the mitochondrial enzyme, ATP synthase F1 subunit beta (ATP5B). The third cluster seems to be mainly related to purine metabolism and redox-detox activities and contains several quinoid dihydropteridine reductases (Qdprb2), an A2ML, and a nucleoside diphosphate kinase (Nme2b.1). The remaining large cluster contains most proteins present in the subnetwork. These are mainly concerned with protein homeostasis and can be placed into subclusters by type. The subclusters include eight forms of the cytoskeletal protein, actin (ACTG2, Acta2, two forms of Actc1a, Acta1a, Actc1, Actc1b and Acta1b), which are involved in cell structure and motility and intracellular vesicle trafficking, three forms of eukaryotic translation elongation factor alpha subunit (Eef1a1a, Eef1a1b, Eef1a2), four Hsps (Hsp70, Hsp70.1, Hsp8, Hspa8b), three forms of ubiquitin (UBC, Uba52, Rps27a), proteins that modulate Hsps and coordinate elimination of damaged/unfolded proteins and protein aggregates via the 26S proteasome, and two protein disulfide isomerase family members (Pdia3 and P4hb). Remaining proteins not assigned to subclusters are ceruloplasmin (Cp), which is engaged in iron transport, 5-aminoimidazole-4-carboxamide ribonucleotide formyltransferase/IMP cyclohydrolase (Atic), which is engaged in purine biosynthesis, and S-adenosylhomocysteine hydrolase (Ahcy), which is engaged in hydrolysis of methionine (Fig. 5a. Right Panel, PPI network enrichment value *P* = 5.95 × 10^− 9^).

When the 74 differentially regulated proteins with significant differences in abundance between *vtg3*-KO and Wt eggs were submitted separately (Wt; *N* = 21, *vtg3*-KO; *N* = 53) to the STRING protein-protein interactions network analysis by mapping to the public zebrafish protein database, they resolved networks with significantly and substantially greater numbers of known and predicted interactions between proteins than would be expected from the same size lists of proteins randomly chosen from the zebrafish database (Fig. 5b). The subnetwork revealed by proteins down-regulated in *vtg3*-KO eggs (Wt; *N =* 21) is made up of a Vtg (Vtg3) weakly interacting with a cluster of five interacting proteins. Proteins in this cluster include three strongly interacting proteins, an enolase involved in glucose metabolism (Eno3), a heat shock protein (Hspa5), and a eukaryotic translation elongation factor alpha subunit (Eef1a1|), two of which (Hsp5 and Eef1a1|1) interact with a catalase (Cat) that, in turn reacts with Crp2 (Fig. 5b. Left Panel; PPI network enrichment value *P* = 6.99 × 10^− 6^).

The subnetwork revealed by proteins up-regulated in *vtg3*-KO eggs (*vtg3*-KO; *N =* 53) is made up of two type-I Vtgs (Vtg5 and Vtg7) that interact with three protein clusters. Two of these clusters are made up of proteins concerned with energy metabolism, the first including two forms of lactate dehydrogenase (Ldhba and Ldha) plus malate dehydrogenase (Mdh2), and the second containing three forms of creatinine kinase (Ckma, Ckmb and Ckba). The third, largest, cluster contains proteins concerned with protein homeostasis that can be arranged as subclusters of proteins by type. These subclusters include seven forms of actin (Acta2, ACTC1, Acta1a, Acta1b, Actc1a, Actc1b and ZGC:86709, a form of Actc2), three Hsps (HSPA8, Hspa8, and Hsp70), three forms of ubiquitin (Uba52, rps27a, and UBC), and three protein disulfide isomerases (Pdia3, Pdia4 and P4hb) (Fig. 5b. Right Panel; PPI network enrichment value *P* = 0.0517).

### STRING networks enrichment analysis

Results of a STRING enrichment analysis of differentially abundant proteins also forming the resolved protein-protein interaction networks in Wt eggs (Wt; *N* = 32) and in *vtg1*-KO (*vtg1*-KO; *N* = 94) eggs for Kyoto Encyclopedia of Genes and Genomes (KEGG) Pathways and Reactome Pathways are given in Table 1. Results for other terms such as the UniProt Keywords, Protein Families Database (PFAM) Protein Domains, and Interpro Protein Families Database (INTERPRO) Protein Domains and Features and Simple Modular Architecture Research Tool (SMART) Protein domains are additionally reported in Table S5. A single KEGG pathway (Arginine and proline metabolism) and a single Reactome pathway (Creatine metabolism) were found to be enriched in proteins down-regulated in *vtg1-*KO eggs (*p* *<* 0.05). KEGG pathways that were enriched in the subnetwork resolved by proteins up-regulated in *vtg1*-KO eggs were; Cysteine and methionine metabolism, Cardiac muscle contraction, Protein processing in endoplasmic reticulum, Metabolic, Folate biosynthesis, Pyruvate metabolism, RNA transport, Andrenergic signaling of cardiomyocytes, Drug metabolism, and Glycolysis/Gluconeogenesis (*p* < 0.05). The corresponding Reactome pathways were Phenylalanine and tyrosine catabolism, AUFI (hnRNP D0) binds and destabilizes mRNA, Attenuation phase, HSP90 chaperone cycle for steroid hormone receptors, Neutrophil degranulation, Immune system, Regulation of HSFI-mediated heat shock response, Metabolism, Cellular response to stress, Innate immune system, Intrinsic pathway of fibrin clot formation, Common pathway of fibrin clot formation, and Calnexin/calreticulin cycle (*p* *<* 0.01).

**Table 1:** KEGG and Reactome Pathways revealed by Network enrichment analyses for differentially regulated proteins which were resolved in a STRING subnetwork in the *vtg1*-KO Experiment. A) proteins down-regulated in *vtg1*-KO eggs. B) proteins up-regulated in *vtg1*-KO eggs. Only statistically significant results are reported (χ^2^, *p* < 0.05). See Table S5 for full analysis report

**A)** ***vtg1*-KO Experiment: Network stats for proteins down-regulated in** ***vtg1*-KO eggs (Wt;** ***N*** **= 32)**			
number of nodes:	17		
number of edges:	18		
average node degree:	2.12		
clustering coefficient:	0.64		
expected number of edges:	6		
PPI enrichment p-value:	2.75E-05		
confidence level:	0.15		
*KEGG Pathways*			
**pathway**	**description**	**protein count**	**FDR**
dre00330	Arginine and proline metabolism	2 of 58	0.0074
*Reactome Pathways*			
**pathway**	**description**	**protein count**	**FDR**
DRE-71288	Creatine metabolism	2 of 10	0.0001
**B)** ***vtg1*-KO Experiment: Network stats for proteins up-regulated in** ***vtg1*-KO eggs (*vtg1*-KO;** ***N*** **= 94)**			
number of nodes:	63		
number of edges:	107		
average node degree:	3.4		
clustering coefficient:	0.387		
expected number of edges:	58		
PPI enrichment p-value:	5.95E-09		
confidence level:	0.40		
*KEGG Pathways*			
**pathway**	**description**	**protein count**	**FDR**
dre00270	Cysteine and methionine metabolism	3 of 47	0.0072
dre04260	Cardiac muscle contraction	3 of 89	0.0135
dre04141	Protein processing in endoplasmic reticulum	4 of 176	0.0135
dre01100	Metabolic pathways	10 of 1278	0.0135
dre00790	Folate biosynthesis	2 of 26	0.0135
dre00620	Pyruvate metabolism	2 of 41	0.0244
dre03013	RNA transport	3 of 151	0.0263
dre04261	Adrenergic signaling in cardiomyocytes	3 of 180	0.0368
dre00983	Drug metabolism - other enzymes	2 of 61	0.0368
dre00010	Glycolysis / Gluconeogenesis	2 of 74	0.0439
*Reactome Pathways*			
**pathway**	**description**	**protein count**	**FDR**
DRE-71182	Phenylalanine and tyrosine catabolism	3 of 14	0.003
DRE-450408	AUF1 (hnRNP D0) binds and destabilizes mRNA	4 of 54	0.003
DRE-3371568	Attenuation phase	3 of 19	0.003
DRE-3371497	HSP90 chaperone cycle for steroid hormone receptors	3 of 21	0.003
DRE-6798695	Neutrophil degranulation	7 of 490	0.0088
DRE-168256	Immune System	11 of 1379	0.0154
DRE-3371453	Regulation of HSF1-mediated heat shock response	3 of 69	0.0238
DRE-1430728	Metabolism	12 of 1751	0.0258
DRE-2262752	Cellular responses to stress	5 of 321	0.0313
DRE-168249	Innate Immune System	8 of 875	0.0313
DRE-140837	Intrinsic Pathway of Fibrin Clot Formation	2 of 19	0.0313
DRE-140875	Common Pathway of Fibrin Clot Formation	2 of 23	0.0365
DRE-901042	Calnexin/calreticulin cycle	2 of 25	0.0387

STRING networks enrichment analysis of differentially regulated proteins also forming the resolved protein-protein interaction networks in Wt eggs (Wt; *N* = 21) and in *vtg3*-KO eggs (*vtg3*-KO; *N =* 53) for KEGG Pathways and Rectome Pathways are given in Table 2. Results for other terms such as UniProt Keywords, PFAM Protein Domains, INTERPRO Protein Domains and Features and SMART Protein domains are additionally reported in Table S6. A single KEGG pathway, Carbon metabolism, was found to be enriched in the network revealed by proteins down-regulated in *vtg3*-KO eggs (Wt; *N =* 21) (*p* < 0.05). KEGG pathways that were enriched in the subnetwork resolved by proteins up-regulated in *vtg3*-KO eggs were (*vtg3*-KO; *N =* 53) Protein processing in endoplasmic reticulum, Cysteine and methionine metabolism, Pyruvate metabolism, Arginine and proline metabolism, Cardiac muscle contraction, Metabolic, Spliceosome, Propanoate metabolism, Andrenergic signaling of cardiomyocytes, Glycolysis/Gluconeogenesis, Endocytosis, MAPK signaling and Ribosome (*p* < 0.05). The corresponding Reactome pathways were AUFI (hnRNP D0) binds and destabilizes mRNA, Attenuation phase, HSP90 chaperone cycle for steroid hormone receptors, Creatine metabolism, Regulation of HSFI-mediated heat shock response, Cellular response to stress, Pyruvate metabolism and citric acid (TCA) cycle, Digestion of dietary carbohydrate, Metabolism of amino acids and derivatives, Calnexin/calreticulin cycle, Pyruvate metabolism, Immune system, Striated muscle contraction, and Hedgehog ligand biosynthesis (*p* *<* 0.01) (Table 2).

**Table 2:** KEGG and Reactome Pathways revealed by Network enrichment analyses for differentially regulated proteins which were resolved in a STRING subnetwork in the *vtg3*-KO Experiment. A) proteins down-regulated in *vtg3*-KO eggs. B) proteins up-regulated in *vtg3*-KO eggs. Only statistically significant results are reported (χ^2^, *p* < 0.05). See Table S6 for full analysis report

**A)** ***vtg3*-KO Experiment: Network stats for proteins down-regulated in** ***vtg3*-KO eggs (Wt;** ***N*** **= 21)**			
number of nodes:	11		
number of edges:	7		
average node degree:	1.27		
clustering coefficient:	0.394		
expected number of edges:	1		
PPI enrichment p-value:	6.99E-06		
confidence level:	0.4		
*KEGG Pathways*			
**pathway**	**Description**	**Protein counts**	**FDR**
dre01200	Carbon metabolism	2 of 125	0.0147
**B)** ***vtg3*-KO Experiment: Network stats for proteins up-regulated in** ***vtg3*-KO eggs (*vtg3*-KO;** ***N*** **= 53)**			
number of nodes:	36		
number of edges:	46		
average node degree:	2.56		
clustering coefficient:	0.417		
expected number of edges:	36		
PPI enrichment p-value:	0.0517		
confidence level:	0.4		
*KEGG Pathways*			
**pathway**	**description**	**protein count**	**FDR**
dre04141	Protein processing in endoplasmic reticulum	6 of 176	3.04E-06
dre00270	Cysteine and methionine metabolism	4 of 47	6.54E-06
dre00620	Pyruvate metabolism	3 of 41	0.00019
dre00330	Arginine and proline metabolism	3 of 58	0.00037
dre04260	Cardiac muscle contraction	3 of 89	0.001
dre01100	Metabolic pathways	8 of 1278	0.001
dre03040	Spliceosome	3 of 131	0.0022
dre00640	Propanoate metabolism	2 of 31	0.0022
dre04261	Adrenergic signaling in cardiomyocytes	3 of 180	0.0041
dre00010	Glycolysis / Gluconeogenesis	2 of 74	0.0091
dre04144	Endocytosis	3 of 293	0.013
dre04010	MAPK signaling pathway	3 of 359	0.0206
dre03010	Ribosome	2 of 126	0.0206
*Reactome Pathways*			
**pathway**	**description**	**protein count**	**FDR**
DRE-450408	AUF1 (hnRNP D0) binds and destabilizes mRNA	5 of 54	5.35E-06
DRE-3371568	Attenuation phase	4 of 19	5.35E-06
DRE-3371497	HSP90 chaperone cycle for steroid hormone receptors (SHR)	4 of 21	5.35E-06
DRE-71288	Creatine metabolism	3 of 10	3.53E-05
DRE-3371453	Regulation of HSF1-mediated heat shock response	4 of 69	0.00014
DRE-2262752	Cellular responses to stress	6 of 321	0.0002
DRE-71406	Pyruvate metabolism and Citric Acid (TCA) cycle	3 of 55	0.002
DRE-189085	Digestion of dietary carbohydrate	2 of 11	0.0037
DRE-71291	Metabolism of amino acids and derivatives	4 of 249	0.0076
DRE-901042	Calnexin/calreticulin cycle	2 of 25	0.0111
DRE-70268	Pyruvate metabolism	2 of 31	0.0157
DRE-168256	Immune System	7 of 1379	0.0343
DRE-390522	Striated Muscle Contraction	2 of 54	0.0372
DRE-5358346	Hedgehog ligand biogenesis	2 of 58	0.0408

### Proteins with significant differences in abundance between *vtg-*KO and Wt eggs

**Fig. 6:**
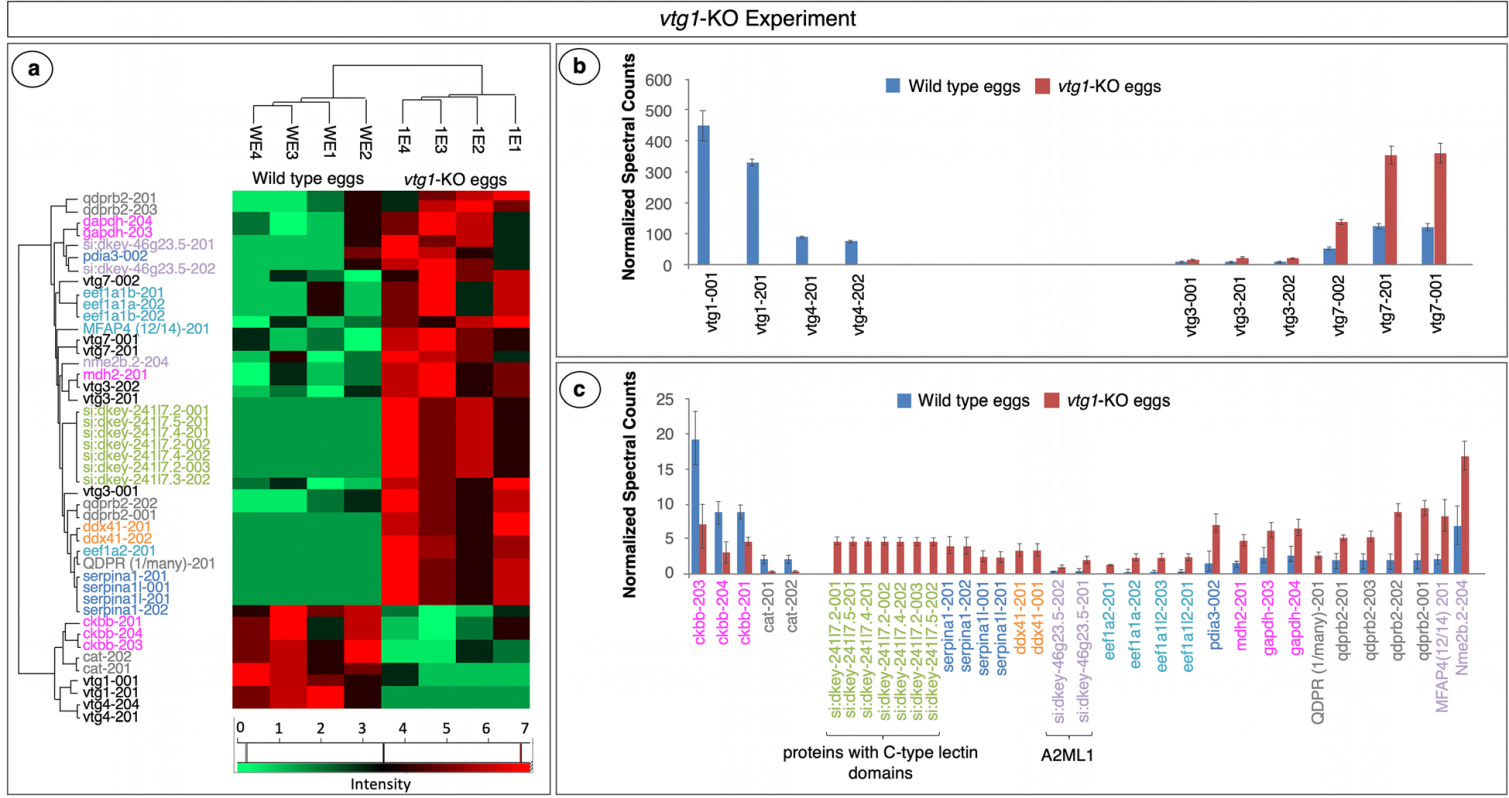
Proteins with significant differences in abundance in *vtg1*-KO Experiment. Panel a. HeatMap representation of differences in abundance based on normalized spectral counts. Panel b. Vitellogenins with significant differences in abundance between *vtg1*-KO eggs and Wt eggs. Panel c. Other proteins with significant differences in abundance between *vtg1*-KO eggs and Wt eggs. All proteins are named for the transcript(s) to which spectra were mapped; for full protein names, see Table S1. Only proteins that were identified in > 4 samples were included in this analysis (t-test, *p* < 0.05, followed by Benjamini Hochberg correction for multiple tests, *p* < 0.05). Vertical bars indicate mean N-SC values (*N* = 4 per group) and vertical brackets indicate SEM. Protein (transcript) labels are color-coded to indicate functional categories to which the proteins were attributed (Fig. 1)

The list of total identified proteins in the *vtg1*-KO experiment (*N* = 301) and in the *vtg3*-KO experiment (*N* = 238) were subjected to filtering by Perseus 1.5.5.3 (available online at https://maxquant.org/perseus/) for candidates to further analyze for statistically significant differences in abundance. No fold difference was taken into account at this step of analysis, but only proteins which were detected in > 4 samples (*N* = 132 for *vtg1*-KO and *N* = 102 for *vtg3*-KO) were carried further for statistical analyses of abundances based on N-SC (t-test followed by Benjamini-Hochberg correction for multiple testing, *p* < 0.05). A total of 45 proteins displayed statistically significant differences in abundance between *vtg1*-KO eggs and Wt eggs (Fig. 6a). Two variants of Vtg1 and two variants of Vtg4 were found to be uniquely identified in Wt eggs and variants of Vtg7 and Vtg3 were 3x and 1.8x more abundant, respectively, in *vtg1*-KO eggs (*p* < 0.05) (Fig. 6b). Three forms of a brain type creatine kinase (Ckbb) and two variants of Cat were significantly less abundant in *vtg1*-KO eggs in comparison to Wt eggs (*p* < 0.05). Among other differentially regulated proteins with significant differences in abundance between *vtg1*-KO eggs and Wt eggs were a nucleoside diphosphate kinase (Nme2b), a microfibril-associated protein 4 (MFAP4), several variants of quinoid dihydropteridine reducatase (Qdprb), two variants of glyceraldehyde-3-phosphate dehydrogenase (Gapdh), a malate dehydrogenase 2 (Mdh2), a protein disulfide-isomerase (Pdia3), several eukaryotic translation elongation factor alpha subunits (Eef1a), two variants of si:dkey-46 g23.5 (predicted to have endopeptidase inhibitor activity), two variants of DEAD (Asp-Glu-Ala-Asp) box polypeptide 41 (Ddx41), two variants of serine (or cysteine) proteinase inhibitor, clade A (alpha-1 antiproteinase, antitrypsin), member 1, like (Serpina1), and many variants of a protein predicted to contain a C-type lectin domain (Fig. 6c).

**Fig. 7:**
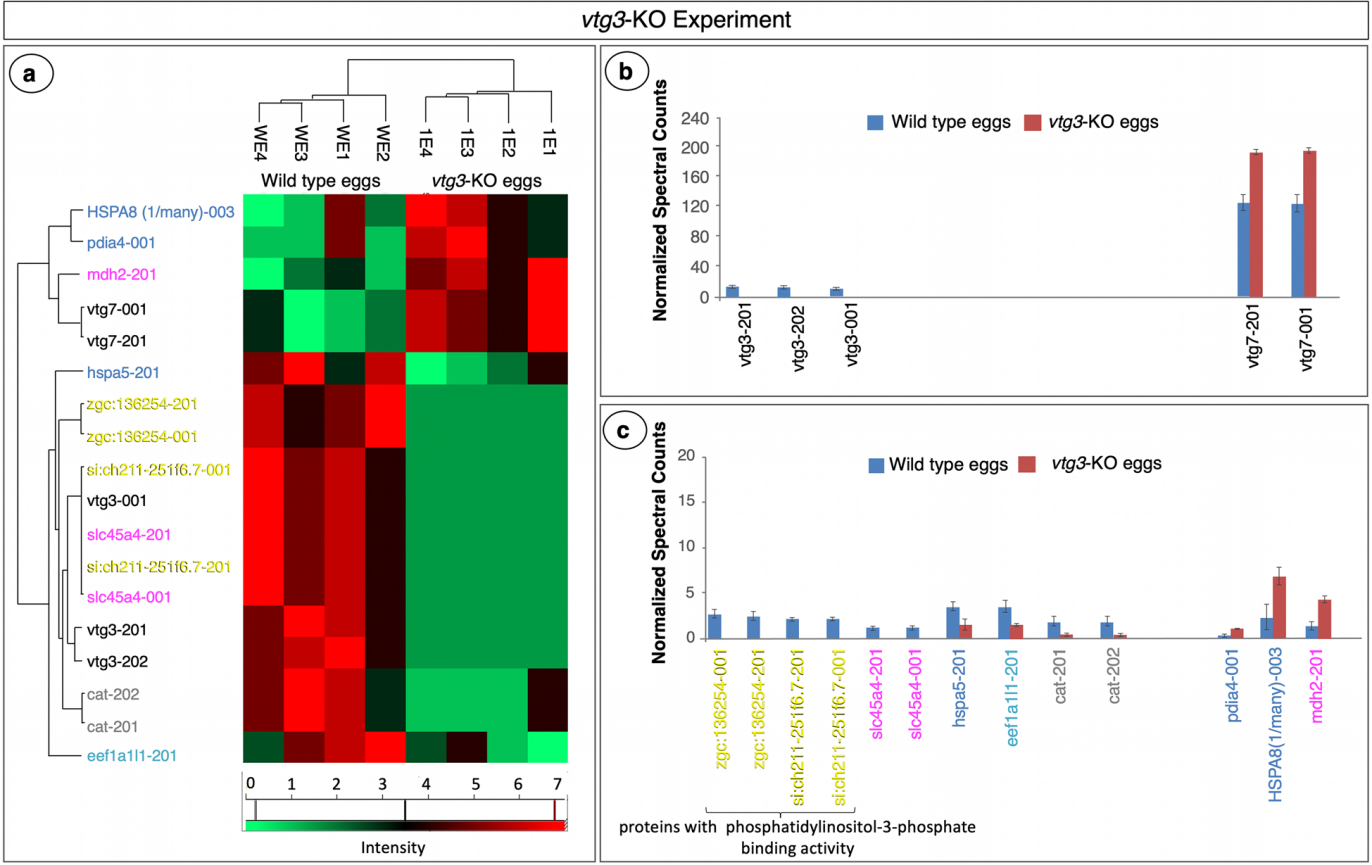
Proteins with significant differences in abundance in the *vtg3*-KO Experiment. Panel a. HeatMap representation of differences in abundance based on normalized spectral counts. Panel b. Vitellogenins with significant differences in abundance between *vtg3*-KO eggs and Wt eggs. Panel c. Other proteins with significant differences in abundance between *vtg3*-KO eggs and Wt eggs. All proteins are named for the transcript(s) to which spectra were mapped; for full protein names, see Table S2. Only proteins that were identified in > 4 samples were included in this analysis (t-test, *p* < 0.05, followed by Benjamini Hochberg correction for multiple tests, *p* < 0.05). Vertical bars indicate mean N-SC values (*N =* 4 per group) and vertical brackets indicate SEM. Protein (transcript) labels are color-coded to indicate functional categories to which the proteins were attributed (Fig. 1)

The exact same series of statistical analysis revealed 18 proteins with significant differences in abundance (N-SC) between *vtg3*-KO eggs and Wt eggs (Fig. 7a). Three variants of Vtg3, two isoforms of si:ch211-251f6.7 and two isoforms of zgc:136254, proteins predicted to be autophagosome/lysosome components with phosphatidylinositol-3-phosphate binding activity in zebrafish (UniprotKB-F1Q1D9_DANRE and UniProtKB Q1RMB1_DANRE), and two variants of solute carrier family 45 member 4 protein (Slc45a4) were found to be uniquely present in Wt eggs (Fig. 7b and c), while Hspa5, a Eukaryotic translation elongation factor 1 alpha 1 like 1 (Eef1a1l1), and two variants of Cat were significantly lower in abundance in *vtg3*-KO versus Wt eggs (Fig. 7c). Two Vtg7 variants, a malate dehydrogenase 2 (Mdh2), a heat shock 70 kDa protein 8 (HSPA8), and a variant of protein disulfide-isomerase (Pdia4) were found to be expressed in significantly higher abundance in *vtg3*-KO eggs than in Wt eggs (*p* < 0.05) (Fig. 7b and c). Of these, Vtg7 was more abundant (1.5-fold) in *vtg3*-KO eggs, just as it was in *vtg1*-KO eggs, potentially compensating for the missing Vtgs in both experiments. Catalases showed a similar pattern of up-regulation in both the *vtg1*-KO and *vtg3*-KO experiments, increasing in abundance 7.2 x and 4.5 x, respectively (*p* < 0.05). Mdh2 was also less abundant in *vtg-*KO eggs in both the *vtg1*-KO experiment (3.6 x) and the *vtg3*-KO experiment (3.4 x) (*p* < 0.05). Finally, two protein disulfide-isomerases (Pdia3 and Pdia4) seem to be upregulated in *vtg1*-KO eggs and in *vtg3*-KO eggs in comparison to Wt eggs with 4.5-fold and 4.3-fold differences, respectively (*p* < 0.05) (Figs. 6 and 7).

## Discussion

### Overview of the biological status and multiple vitellogenin content of *vtg*-KO zebrafish eggs

Our previous studies involving knock-out (KO) of *vtg* genes using CRISPR/Cas9 technology demonstrated that certain type-I Vtgs (Vtg1, 4, and 5) and type-III Vtg (Vtg3) are essential for egg quality and developmental competence in zebrafish [33, 34]. The present study was undertaken to gain insight into the molecular mechanisms by which KO of these *vtg* genes influences reproductive processes at the proteomic level, representing the dynamic transfer of genetic information into the actual effector molecules in the cell.

In order to better understand the results of this research it is important to review the status of the *vtg*-KO eggs in terms of Vtg repertoire and cellular processes that occur at 1-cell stage after fertilization. To begin, it is critical to note that no highly active protein synthesis and mitochondrial replication is expected to occur within the first hour of development after fertilization in zebrafish embryo that is still undergoing the first cellular division and wherein most cellular functions and energy homeostasis are entirely dependent on maternal cargo and mitochondrial pool. In our previous study, it was proven that *vtg1*-KO eggs, which are used in this study, do not contain Vtg1, 4 and 5, while they are still rich in Vtg6, especially Vtg7, Vtg2 and Vtg3, and *vtg3*-KO eggs do contain all type I and type II Vtgs while they lack Vtg3, the least abundant type of Vtg in the egg, only [33]. Offspring mortality in *vtg1*-KO line occurred by 5 dpf at which stage the embryo are dependent to highly active protein and energy homeostasis derived from embryonic genome activities [33]. The increased number of eggs produced by *vtg1*-KO females was interpreted as a form of adaptation to compensate the lower fertility and thus to ensure the number of surviving offspring [33]. However, despite these compensatory efforts of the mother, the dramatic drop in offspring survival even after embryonic genome activation was unavoidable and intriguing [33]. Apart from structural deformities, such as cardiac and yolk sac edema which is accompanied by mobility and feeding issues, this failure points to the loss of vital substrates caused by removing Vtg1, 4 and 5 [33]. Likewise, in addition to substantial reduction in egg fertilization rates the absence of Vtg3 causes fatal developmental impairments and massive mortalities in offspring from *vtg3*-KO line at a stage as early as 8 hpf, still after the embryonic genome activation, which is even more astonishing [33].

In salmonids, vitellogenins may account for 90% of the final oocyte volume [35]. Link et al., [36] observed an over 10-fold decrease in total protein content in zebrafish embryos after deyolking at 3 1/3 hpf stage which evinces a 94% yolk to embryo protein content ratio. Type-I Vtgs, constituting 84% of the egg yolk and ~ 90% of the total protein in zebrafish eggs were expected to nourish all cellular activities at early stages of embryonic development, during the meroblastic stage. Despite a ~ 3 fold significant increase in Vtg7 abundance, invalidation of *vtg1,4* and *5* resulted in a 50% reduction in the overall amount of type-I Vtgs in *vtg1*-KO eggs. These modifications could have major impact on the dynamics of cellular homeostasis in *vtg1*-KO eggs. The *vtg3*-KO egg contained all type I and type II Vtgs as well as a ~ 1.5-fold significant increase in Vtg7 levels. Despite the cell’s best efforts to compensate for the loss of one Vtg with another for the sake of survival, the absence of any Vtg invalidated in this study still leads to substantial mortality in zebrafish embryos/larvae. As a result, each form of Vtg appears to have specific and critical functions during zebrafish development.

### *vtg*-KO caused changes in proteomic profiles of zebrafish eggs

In 1-cell stage zebrafish embryos incapacitation of *vtg1*, *4*, and *5,* or of *vtg3,* divulged highly significant changes in the proteomic profiles, and thus, in the abundance of proteins involved in energy homeostasis, protein synthesis and cellular proliferation. In both *vtg1*-KO and *vtg3*-KO eggs, the findings of frequency distribution analysis agreed with those of PANTHER overrepresentation tests, attributing a much higher proportion of proteins to GO terms related to protein degradation and synthesis inhibition and to cell cycle, division, growth and fate. The STRING network and network enrichment analyses revealed that proteins linked to these same activities, as well as activities involving energy metabolism were enriched in *vtg*-KO egg. These findings are puzzling, particularly because *vtg1*-KO eggs still contain Vtg2, Vtg3, Vtg6, and Vtg7, and the *vtg3*-KO eggs still contain all type-I and type-II Vtgs.

#### Protein homeostasis and ER functions

Embryogenesis follows an evolutionary conserved stereotypical procedure and results in functional tissues and organs [37]. During this process cellular functions are reliant on proteostasis which requires appropriate regulation of protein synthesis, protein folding, and protein degradation [38]. The early stages of embryonic development proceed with no new transcription and with minimal de novo protein synthesis in many species, including African clawed frog (*Xenopus laevis)*, Mongolian gerbil (*Meriones unguiculatus)*, and sheep (*Ovis aries*) [39–41]. In *Xenopus* ribosomal genes are not transcribed until the late gastrula stage, when the embryo has reached nearly 10,000 cells [42], and this situation is likely to occur in a similar manner in fish [43]. As a result, the embryo must either unmask existing RNA for translation or modify existing proteins in order to sustain ongoing cellular activities during the first cleavage stages [44].

Correct protein folding is a key step in protein synthesis. Accumulation of misfolded and/or unfolded proteins in the ER lumen causes its malfunction, leading to ER stress. The ERQC (ER quality control system) is in charge of identifying properly folded proteins versus misfolded proteins [45, 46]. ERQC approved proteins are channeled for transport to the Golgi complex, while misfolded proteins are retained in the ER to undergo correct folding or to be targeted for proteolysis by the ER degradation (ERAD) machinery [45, 46]. Unfolded protein response (UPR) is a cascade of adaptive pathways that seeks to preserve cellular homeostasis and normal ER function in response to ER stress. However, the UPR is incapable of restoring ER homeostasis and normal function in the event of severe or prolonged ER stress, which in turn causes activation of apoptosis [45–48]. The exact level of protein synthesis that occurs in early stages of zebrafish embryonic development is unknown. However, the differential abundance of proteins related to protein degradation and synthesis inhibition, and overrepresentation of proteins related to biological processes such as chaperone-mediated protein folding, cellular response to unfolded and/or topologically incorrect proteins, and vesicle-mediated transport, in both types of *vtg*-KO eggs, can point to ER stress and activated UPR in the absence of these certain types of Vtgs.

Heat shock proteins (e.g. Hsp70 kDa) and protein disulfide-isomerase (Pdi) are multifunctional ER-resident redox chaperones [48] that are activated under ER stress conditions [49]. They are responsible for the isomerization, formation, and rearrangement of protein disulphide bonds in highly specific manner, providing a mechanism to maintain native protein conformation and thus protecting the cell against protein misfolding. As evidenced by the STRING network analyses, overexpression of protein disulphide isomerases (Pdia3, Pdia4 and P4hb), Hsp70 and other Hsp70-family proteins (Hsp70.1, Hspa), and ubiquitins (UBC, Uba52, and Rps27a), key regulators of protein turnover and protein targeting for proteasomal degradation, in both types of *vtg*-KO eggs, can be indicators of ER stress related disorders. Furthermore, overexpression of multiple forms of redox/detox activity associated proteins (Qdprs) in *vtg3*-KO eggs, adds to the evidence that *vtg*-KO eggs are likely to be under ER stress.

#### Energy homeostasis and mitochondrial functions

Under normal circumstances fertilized eggs, which initiate a series of cell divisions within 45 min after fertilization in zebrafish, are operated and/or fueled by maternal transcripts, proteins, lipids and other key molecules loaded into the egg. These activities require, apart from cellular proteostasis, properly functioning cell cycle machinery and high energy to conduct. In addition to ATP production, mitochondria perform a number of functions that are vital for embryo survival, including intracellular calcium sequestration and release, free fatty acid synthesis, regulation of cell death and serving as a repository of intact mitochondrial DNA (mtDNA) for developing offspring [50, 51]. Deficiencies in mitochondrial function and mtDNA number have been shown to impact egg quality and developmental competence in different yet overlapping ways [52, 53]. It has been proposed that the total content of mtDNA in zebrafish, as in mammals, is reliant on maternal reservoirs [54, 55]. Before embryonic mitochondria take over, the embryo is dependent on the functioning of the existing maternal mitochondria supply to provide the required energy for viability [56, 57]. Embryonic mitochondrial replication does not occur until the blastocyst stage in mammals, which corresponds to the mid-blastula stage in zebrafish, when the embryonic genome is activated.

Despite a stagnation in mitochondrial proliferation prior to zygotic genome activation, the early embryo still needs oxidative phosphorylation by mitochondria to provide ATP [52, 58]. Oxidative metabolism is present even in the unfertilized eggs and continues throughout early development in rainbow trout [59]. The limited number of mitochondria in early zebrafish embryos are active and existing free fatty acids (FFAs) are reported to serve as the substrate for oxidative phosphorylation to supply the required ATP [60]. FAAs, whose carbon skeletons are fed into the tricarboxylic acid cycle, are known to fuel intermediary metabolism [61] and to support gluconeogenesis, producing glucose as an important substrate for synthesis of nucleic acids and polysaccharides in later embryonic development [62]. FAAs derived from Vtgs, which constitute the majority of the maternal cargo in eggs, are produced in zebrafish and possibly all teleosts via an intensive secondary proteolysis of yolk proteins [63]. This secondary proteolysis is primarily a mechanism for generation of FAA osmolytes that promote oocyte hydration during final oocyte maturation, and that also are a critical source of substrates for energy generation and other vital developmental activities taking place in the egg and early embryo. These latter activities may become more prominent in *vtg*-KO embryos because proteins up-regulated in both *vtg1*-KO and *vtg3-*KO eggs are overrepresented in the cysteine and methionine metabolism, protein processing in endoplasmic reticulum, pyruvate metabolism, and glycolysis/gluconeogenesis KEGG pathways (Tables 1 and 2). Furthermore, a significantly lower abundance of Slc45a4 variants, which are thought to be involved in regulation of glucose homeostasis [64] and concomitant increase of Mdh2 in both *vtg1*-KO and *vtg3*-KO eggs contribute to the description of a proteome underpinning disturbed energy homeostasis in *vtg*-KO eggs (see Figs. 6 and 7). Additional significant increase in Gapdh and the decrease of Ckbb in *vtg1*-KO eggs is strengthening the conjecture of this potential disturbance.

Differential regulation of several other proteins that are involved in redox-detox activities (five Qdprb2 variants, Nme26.1, A2ML), forming part of a cluster resolved by the STRING network analysis of *vtg1*-KO eggs (Fig. 5), and the significantly lower abundance of Catalase variants, important enzymes protecting the cell from oxidative damage by reactive oxygen species (ROS), in both *vtg1*-KO and *vtg3*-KO eggs (Figs. 6 and 7), can be indicators of oxidative stress, which induces mitochondrial DNA mutations, damages the mitochondrial respiratory chain, alters membrane permeability, and impedes Ca^+ 2^ homeostasis and mitochondrial defense systems [65].

#### Lipid metabolism

In comparison to Wt eggs, *vtg3*-KO eggs are significantly deficient in proteins related to lipid metabolism and variants of two proteins (zgc:136254 and si:ch211-251f6.7) that are predicted to have phosphatidylinositol-3-phosphate binding activity exhibit significantly lower abundance in *vtg3*-KO eggs (Fig. 7c). These proteins are also predicted to be present on the autophagosome and lysosomal membranes. In contrast to what has previously been stated for oviparous vertebrates, Dutta and Sinha [60] have demonstrated that lipid droplets, rather than yolk derived lipids, are the critical embryonic source of FFAs to maintain ATP homeostasis during early developmental stages (0–5 hpf) of zebrafish embryos. This implies that a FFA biosynthesis is ongoing at the early stages of embryonic development in zebrafish. The ER and Golgi apparatus are known to be major sites of production of membrane phospholipids whereas mitochondria are known to be FFA synthesis sites in the cell. The potential ER and mitochondrial dysfunctions discussed above may culminate in abnormalities in FFA biosynthesis and phospholipid metabolism, as seen in *vtg3*-KO eggs. However, despite the interrelation of these molecular changes, surviving *vtg3*-KO offspring did not exhibit obvious problems with mobility and, like Wt offspring, they were swimming and feeding actively, which was not observed for surviving *vtg1*-KO offspring [33]. Being the least abundant form of Vtgs found in all vertebrate eggs, the specific contribution of Vtg3 to cellular functions related to energy and lipid metabolism mechanisms remain unelucidated and needs to be further investigated.

### *vtg*-KO caused changes in biological pathway regulations

PANTHER pathways that were significantly overrepresented by differentially abundant proteins in both *vtg1*-KO and *vtg3*-KO zebrafish eggs (Fig. 4) paint a clear portrait of impairments that could be caused by the absence of certain Vtgs following *vtg* gene KO. These common pathways were mainly represented by a set of Actins including, Actc1a, Actc1b, Acta2, ACTC1 (1 of many). Actins dominate the subnetwork of differentially regulated proteins from both *vtg1*-KO and *vtg3*-KO eggs revealed by the STRING analyses as well (Fig. 5). Actins and microtubules are major cytoskeletal elements of most cells including neurons. In order for a cell to move and change shape or undergo cell division, its cytoskeleton must undergo rearrangements that involve breaking down and reforming actin filaments. These same elements are required for the vesicle trafficking between cell compartments as well as communication with the internal and external cellular environment, as seen during endocytosis. As example, teleost oocytes grow several orders of magnitude via the uptake of Vtgs through the endocytosis during oocyte growth phase.

Among significantly overrepresented PANTHER pathways, the Cytoskeletal regulation by Rho GTPase pathway revealed the highest fold enrichment and statistical significance for both *vtg1*- and *vtg3*-KO eggs. The Cadherin signaling pathway, perhaps in conjunction with the Nicotinic acetylcholine receptor signaling pathway, exhibits a similar pattern of increased enrichment by differentially regulated proteins in *vtg*-KO eggs (Fig. 4, Tables S3 and S4). In both *vtg1*- and *vtg3*-KO zebrafish eggs differentially abundant proteins significantly overrepresent the inflammation mediated chemokine and cytokine and the Wnt signaling pathways as well (Fig. 4). These findings were further supported by network enrichment analysis results (i.e. significant enrichment in Reactome pathways such as the cellular response to stress, innate immune system, fibrin clot formation and neutrophil degranulation, among others) (Table 1 and Table 2) which may be indicators of inflammatory disorders. The significant increase in Ddx41 abundance in *vtg1*-KO eggs (Fig. 6) supports these findings on the compromised state of the cellular immune system. The Wnt signaling pathway regulates a multitude of cellular processes including cell fate determination, motility, polarity, primary axis formation and organogenesis, and dysregulated Wnt signaling has catastrophic consequences for the developing embryo [66]. Highly significant overrepresentation of the aforementioned pathways in *vtg*-KO zebrafish eggs is most likely related to the aberrant development and function of cardiovascular system, which leads to pericardial edema and inflammatory responses culminating in death. The enrichment of *vtg-*KO eggs by proteins related to the cardiac muscle contraction KEGG pathway revealed by network enrichment analysis (Table 1 and Table 2) as well as previously reported *vtg-*KO zebrafish phenotypes such as pericardial and yolk sac edema [33] suggest that Vtgs do indeed contribute to heart development and function in zebrafish. Overrepresentation of proteins involved in cell cycle, division, growth and fate related activities in *vtg*-KO fish (Fig. 2), as well as differential abundance of MFAP4 and Nme2b.2 in *vtg1*-KO eggs (Fig. 6), may explain the observed phenotypes and massive mortalities during critical early developmental stages of *vtg1*- and *vtg3*-KO zebrafish offspring.

The Alzheimer’s disease-persenilin pathway and the Huntington disease (HD) are human neurodegenerative disease related pathways that are significantly overrepresented by differentially abundant proteins in *vtg*-KO zebrafish eggs. Neurodegenerative diseases are known to be caused by oxidative stress related mitochondrial disorders which mediate or amplify neuronal dysfunction, thereby triggering neurodegeneration [65]. Perturbations in mitochondrial maintenance, localization and activity leading to subsequent impairments in normal development, are thought to be linked to neurodegenerative diseases, such as Alzheimer’s disease (AD), Amyotrophic lateral sclerosis (ALS) and Parkinson’s disease (PD) [67–71]. In addition, recent research suggests a systemic link between cardiovascular disorders and the AD [67]. On the one hand, HD pathogenesis is linked to numerous observations related to mitochondrial dysregulation [72], while on the other hand, it is thought to arise from aberrant protein folding and can thus be regarded as a protein conformational disease [72, 73]. According to Labbadia and Marimoto [72], the chronic expression of misfolded proteins may cause progressive neurotoxicity through common mechanisms and pathways. As previously discussed, a high percentage distribution of proteins related to protein degradation and synthesis inhibition, overrepresentation of biological processes related to protein folding and cellular response to unfolded protein, and significant STRING network clusters labeled as protein homeostasis in Fig. 5a and b are strong evidence of protein folding dysfunctions in *vtg*-KO zebrafish eggs.

Unlike the *vtg1*-KO eggs, differentially abundant proteins in *vtg3*-KO eggs significantly over-represented the apoptosis signaling and Parkinson’s disease PANTHER pathways (Fig. 4). Apoptosis is a type of programmed cell death, that is generally characterized by distinct morphological characteristics and energy-dependent biochemical mechanisms [74], and it is considered to be an essential component of many physiological processes, including embryonic development. Some conditions feature insufficient apoptosis whereas others feature excessive apoptosis [74]. As previously stated, in severe ER stress conditions, the cell may be unable to re-establish ER homeostasis and normal functioning, leading to apoptosis [45–48]. Furthermore, any failure of mitochondrial quality control could prevent removal of damaged mitochondria, facilitating apoptosis to occur during time of high cellular stress. Based on the substantial proteomic changes that resulted, it appears certain that the absence of knocked out Vtgs induces severe cellular stress on the way to the induction of the observed developmental disorders [33]. The underlying mechanisms by which Vtg3 forestalls these disorders, however, remain unclear.

### Similarities in *vtg*-KO and poor quality driven proteomic profiles in zebrafish eggs

The proteomic profiles of *vtg*-KO zebrafish eggs are strikingly similar to those of poor quality zebrafish eggs previously reported [34]. Several C-type lectin domain-containing protein variants (si:dkey241l7) were significantly increased in abundance in *vtg1*-KO eggs. C-type lectins have been found in the cortical granules of fish eggs, from whence they are released during fertilization into the perivitelline space, where they assist in water hardening of the egg and prevention of polyspermy [75]. Although the exact function of these lectins and their relationship to the absence of Vtgs has yet to be determined, the disparate expression of these proteins in zebrafish eggs of different quality grades as well as in *vtg1*-KO eggs, suggests that they may impact embryo development. Similarly, poor quality zebrafish eggs were found to have deficiencies in protein synthesis, energy and lipid metabolisms, a surfeit of proteins involved in endo-lysosomal activities, autophagy and apoptosis, as well as some oncogene products, lectins and egg envelope proteins. Furthermore, PANTHER pathways overrepresentation profiles of poor quality zebrafish eggs revealed the same signatures for human neurodegenerative diseases pathways as is in *vtg-*KO zebrafish eggs. In addition, dysregulation of the Wnt signaling pathway and the Cytoskeletal regulation by Rho GTPase pathways were found in poor quality zebrafish eggs in the same previous study [34]. Poor quality zebrafish eggs were thought to originate from oocytes that did not make it through the final maturation where Vtgs were proteolyzed to free amino acids. And this failure was considered as a possible cause of impaired molecular mechanisms. This hypothesis is fortified by evidence from our current study, which shows that the absence of Vtgs would results in a very similar outcome to oocyte maturation failure.

## Conclusions

This study provides astonishing signatures of molecular changes and subsequent cellular dysfunctions caused by the invalidation of certain Vtgs in zebrafish eggs at proteomic level. These signatures of impairments in the cellular functions, which significantly alter embryonic development even after zygotic genome activation, can be detected as early as the 1-cell stage of the fertilized egg. Accordingly, the lack of Vtg1, 4, and 5 of type I Vtgs in *vtg1*-KO or the Vtg3 in *vtg3*-KO zebrafish eggs appears to have vital impacts on protein and energy homeostasis, which affects cellular proliferation and embryonic developmental competence. Because the majority of maternal supplies and structures (i.e., FAA, FFA, ions and mitochondria) that will feed and foster cellular activities during early embryonic development are generated during oocyte maturation, the overall findings of this study further emphasize the importance of proper Vtg processing and utilization at this stage. The discovery of common molecular signatures in both *vtg1*- and *vtg3*-KO eggs, as well as their similarity to proteomic profiles of poor quality zebrafish eggs, eliminates any doubts on contributions of multiple Vtgs to egg quality and supports the hypothesis of potential oocyte maturation impairments in poor quality eggs and those lacking Vtgs. At this junction, more research is needed to determine the specific role of each zebrafish Vtg in the impaired molecular mechanisms. However, several differentially abundant proteins representing the altered molecular mechanisms have been unveiled and can be considered as strong candidate markers for studying the details of these mechanisms during early embryonic development in zebrafish and potentially other vertebrates.

## Methods

### Animal care and biological sample collection

Pure *vtg1*-KO and *vtg3*-KO zebrafish lines were produced and adults from these lines as well as Wt (non-related wild type) lines maintained and spawned as indicated by Yilmaz et al. [33, 34]. Forty eggs per spawn were collected immediately after fertilization, prior first cellular division, and stored at -80 °C until being used for mass spectrometry analyses.

### Protein extraction and SDS-PAGE

Protein extraction from egg samples and quantification of total protein content in these extracts was performed as previously described [34]. Protein extract (60 μg) were then denatured and run on SDS-PAGE and prepared for in-gel tryptic digestion as indicated by Yilmaz et al. [34] with the exception that the samples were run for only 2 min (until the protein mix entered completely into the stacking gel) and were excised from gel in a single band form carrying all proteins contained in the biological samples after protein fixation and gel staining procedures [34].

### In-gel tryptic digestion and LC-MS/MS

Following repeated washes in MilliQ water, gel pieces were prepared for in-gel tryptic digestion via de-coloration followed by alkylation and reduction and subjected to in-gel tryptic digestion as previously described by Yilmaz et al. [34]. Protein digests were recovered in several steps and evaporated to dryness in a vacuum centrifuge. Pellets containing digested peptides were then resolubilized in 30 μl of 95% H_2_O: 5% Formic acid by vortex mixing for 10 min before being subjected to LC-MS/MS.

Peptide mixtures were analyzed with a nanoflow high-performance liquid chromatography (HPLC) system (LC Packings Ultimate 3000, Thermo Fisher Scientific, Courtaboeuf, France) connected to a hybrid LTQ-OrbiTrap XL spectrophotometer (Thermo Fisher Scientific) equipped with a nanoelectrospray ion source (New Objective), as previously described [76]. The mass spectrometer was operated in the data-dependent mode by automatic switching between full-survey scan MS and consecutive MS/MS acquisition. Survey full scan MS spectra (mass range 400–2000) were acquired in the OrbiTrap section of the instrument with a resolution of r = 60,000 at m/z 400; ion injection times are calculated for each spectrum to allow for accumulation of 10^6^ ions in the OrbiTrap. The ten most intense peptide ions in each survey scan with an intensity above 2000 were sequentially isolated and fragmented in the linear ion trap by collision-induced dissociation. For OrbiTrap measurements, an external calibration was used before each injection series ensuring an overall error mass accuracy below 5 ppm for the detected peptides. MS data were saved in RAW file format (Thermo Fisher Scientific) using XCalibur 2.0.7 with tune 2.4.

The mass spectrometry proteomics data have been deposited to the ProteomeXchange Consortium [77] via the PRIDE [78] partner repository under the project name “Egg proteome in *vtg1* and *vtg3* knocked-out zebrafish females” with the dataset accession number PXD021302 and project DOI number of 10.6019/PXD021302

### Protein identification, quantification, annotation and statistics

The spectra search was performed with the Proteome Discoverer 1.2 software supported by Mascot (Mascot server v2.2.07; http://www.matrixscience.com). Obtained MS/MS spectra were searched against a target-decoy concatenated database created from the zebrafish Ensembl proteome database (*Danio rerio*_Zv9, March 2015) using Mascot (Matrix Science) under the condition settings previously described by Yilmaz et al. [34]. Attributed spectra were then analyzed using ProteoIQ 2.8 (Premier Biosoft, Palo Alto, CA, USA) at < 1% FDR, 0.5% minimum protein group probability, and 6 aa minimum peptide length, in order to identify quantify proteins based on their N-SC values. For each protein, obtained spectral counts were normalized in three sequential steps; a) normalization by apportion of shared peptides based on the number of unique peptides each protein group possessed, b) normalization by the total spectral counts between replicates and biological samples, and c) normalization by the size of each protein (amino acid residues).

The list of total identified proteins in both experiments (*N* = 301 and *N* = 238 for *vtg1*-KO and for *vtg3*-KO, respectively) were first filtered manually to exclude those detected in less than 4 biological samples and have less than 1.5 fold difference in abundance and the remaining proteins were annotated using the GO, KEGG and Database for Annotation, Visualization and Integrated Discovery (DAVID) [79–82] functional annotation tools. These proteins were then classified into thirteen arbitrarily chosen functional categories that would account for > 90% of the proteins as originally suggested by Yilmaz et al., [34]. These functional categories are: protein synthesis, energy metabolism, lipid metabolism, cell cycle, division, growth and fate, protein degradation and synthesis inhibition, oxidoreductase (redox)- and detoxification (detox)-related, immune function-related, Lectins and Vtgs. Differentially regulated proteins that could not be attributed to any of these categories and were placed in the category “Other”. For simplicity, proteins were attributed to only one category considered as the ‘best’ fit. Presented results are based on consensus annotations of two independent observers made before any other analyses categorizing the proteins (i.e. observations made ‘blind’). Chi square analysis with significance level of (*p* < 0.05) was used to detect differences between groups in the distribution of differentially regulated proteins among functional categories.

Overrepresentation analyses were conducted using the PANTHER-GO Slim enrichment tool from GO Consortium [83] available online at http://geneontology.org/ for Biological Process, Molecular Function, PANTHER Pathway [84], Protein Class and Cellular Component. Proteins which were differentially regulated in *vtg1*-KO and *vtg3*-KO experiments were subjected to the analysis of protein-protein interaction networks [85] separately using the STRING Network search tool available from the STRING Consortium online at http://string-db.org/, with the data settings Confidence: Medium (0.40), Max Number of Interactions to Show: None/query proteins only. For the PANTHER and STRING analyses, only statistically significant enrichment results (*p* < 0.05) are reported.

All proteins which were identified in > 4 samples (*vtg1*-KO; *N* = 132, *vtg3*-KO; *N* = 102) were additionally analyzed for statistically significant differences in abundance using student’s t-test (*p* < 0.05) followed by Benjamini-Hochberg correction for multiple testing (*p* < 0.05) (IBM SPSS Statistics Version 19.0.0, Armonk, NY).

## Supplementary Information

**Additional file 1.**

## Data Availability

The mass spectrometry proteomics data have been deposited to the ProteomeXchange Consortium via the PRIDE partner repository under the project name “Egg proteome in *vtg1* and *vtg3* knocked-out zebrafish females” with the dataset accession number PXD021302 and project DOI number of 10.6019/PXD021302

## Abbreviations

CRISPR/Cas9: Clustered Regularly Interspaced Short Palindromic Repeats/ CRISPR associated protein 9
KO: Knock-out
Vtg: Vitellogenin
LC-MS/MS: Liquid Chromatography Tandem Mass Spectrometry
*vtg1*-KO: *vtg1,4,5*-knock-out
*vtg3*-KO: *vtg3*-knock-out
Wt: Wild type
hpf: Hours post fertilization
YPs: Yolk Proteins
LvH: Lipovitellin Heavy Chain
LvL: Lipovitellin Light Chain
Pv: Phosvitin
β’-c: β’-component
Ct: C terminal
VtgR: Vitellogenin Receptor
CatD: Cathepsin D
FAAs: Free Amino Acids
CatB: Cathepsin B
CatL: Cathepsin L
N-SC: Normalized Spectral Counts
PANTHER: Protein Analysis Through Evolutionary Relationships
GO: Gene Ontology
STRING: Search Tool for the Retrieval of Interacting Genes/Proteins
ROS: Reactive Oxygen Species
KEGG: Kyoto Encyclopedia of Genes and Genomes
PFAM: Protein Families Database
INTERPRO: Interpro Protein Families Database
SMART: Simple Modular Architecture Research Tool
TCA: Tricarboxylic Acid Cycle
ER: Endoplasmic Reticulum
ERQC: Endoplasmic Reticulum Quality Control
ERAD: ER Degradation
UPR: Unfolded Protein Response
mtDNA: Mitochondrial DNA
FFAs: Free Fatty Acids
HD: Huntington Disease
ALS: Amyotrophic Lateral Sclerosis
AD: Alzheimer Disease
PD: Parkinson’s Disease
HPLC: High Performance Liquid Chromatography
FDR: False Discovery Rate
DAVID: Database for Annotation, Visualization and Integrated Discovery

## References

[1] Hiramatsu N, Hara A, Hiramatsu K, Fukada H, Weber GM, Denslow ND, et al. Vitellogenin-derived yolk proteins of white perch, *Morone americana*: purification, characterization, and vitellogenin-receptor binding. Biol Reprod. 2002;67(2):655–67. 10.1095/biolreprod67.2.655. 10.1095/biolreprod67.2.655 12135911

[2] Finn RN, Kristoffersen BA (2007). Vertebrate vitellogenin gene duplication in relation to the “3R hypothesis”: correlation to the pelagic egg and the oceanic radiation of teleosts. PLoS One.

[3] Reading BJ, Hiramatsu N, Sawaguchi S, Matsubara T, Hara A, Lively MO, et al. Conserved and variant molecular and functional features of multiple vitellogenins in white perch (*Morone americana*) and other teleosts. Mar Biotechnol. 2009;11(2):169–87. 10.1007/s10126-008-9133-6. 10.1007/s10126-008-9133-6 18766402

[4] Wang H, Tan JT, Emelyanov A, Korzh V, Gong Z. Hepatic and extrahepatic expression of vitellogenin genes in the zebrafish, *Danio rerio*. Gene. 2005;356:9–100. 10.1016/j.gene.2005.03.041. 10.1016/j.gene.2005.03.041 15979250

[5] Finn RN, Kolarevic J, Kongshaug H, Nilsen F (2009). Evolution and differential expression of a vertebrate vitellogenin gene cluster. BMC Evol Biol.

[6] Meng X, Bartholomew C, Craft JA (2010). Differential expression of vitellogenin and oestrogen receptor genes in the liver of zebrafish, *Danio rerio*. Anal Bioanal Chem.

[7] Yilmaz O, Patinote A, Nguyen T, Bobe J (2018). Multiple vitellogenins in zebrafish (*Danio rerio*): quantitative inventory of genes, transcripts and proteins, and relation to egg quality. Fish Physiol Biochem.

[8] Patiño R, Sullivan CV (2002). Ovarian follicle growth, maturation and ovulation in teleost fish. Fish Physiol Biochem.

[9] Finn RN (2007). The maturational disassembly and differential proteolysis of paralogous vitellogenins in a marine pelagophil teleost: a conserved mechanism of oocyte hydration. Biol Reprod.

[10] Hiramatsu N, Cheek AO, Sullivan CV, Matsubara T, Hara A. Vitellogenesis and endocrine disruption. In: Mommsen TP, Moon T, editors. Biochemistry and Molecular Biology of Fishes, Environmental Toxicology, vol. 6. Amsterdam: Elsevier Science Press; 2005. p. 431–71. 10.1016/S1873-0140(05)80019-0.

[11] Reading BJ, Sullivan CV. The reproductive organs and processes - Vitellogenesis in Fishes. In: Farrell AP, editor. Encyclopedia of Fish Physiology: Academic Press; 2011. p. 635–46. 10.1016/B978-0-12-374553-8.00257-4.

[12] Sullivan CV, Yilmaz O. Vitellogenesis and yolk proteins, fish. In: Skinner MK, editor. Encyclopedia of Reproduction (Second Edition): Academic Press; 2018. p. 266–77. 10.1016/B978-0-12-809633-8.20567-0.

[13] Babin PJ, Carnevali O, Lubzens E, Schenider WJ. Molecular aspects of oocyte vitellogenesis in fish. In: Babin P, Lubzens E, Cerda J, editors. The Fish Oocyte: From Basic Studies to Biotechnological Applications: Springer; 2007. p. 39–76. 10.1007/978-1-4020-6235-3_2.

[14] Opresko LK, Wiley HS (1987). Receptor-mediated endocytosis in *Xenopus* oocytes: I- characterization of vitellogenin receptor system. J Biol Chem.

[15] Matsubara T, Koya Y (1997). Course of proteolytic cleavage in three classes of yolk proteins during oocyte maturation in barfin flounder, *Verasper moseri*, a marine teleost spawning pelagic eggs. J Exp Zool.

[16] Carnevali O, Centonze F, Brooks S, Marota I, Sumpter JP (1999). Molecular cloning and expression of ovarian cathepsin D in seabream, *Sparus aurata*. Biol Reprod.

[17] Carnevali O, Carletta R, Cambi A, Vita A, Bromage N (1999). Yolk formation and degradation during oocyte maturation in seabream, *Sparus aurata*: involvement of two lysosomal proteinases. Biol Reprod.

[18] Carnevali O, Cionna C, Tosti L, Lubzens E, Maradonna F (2006). Role of cathepsins in ovarian follicle growth and maturation. Gen Comp Endocrinol.

[19] Craik J, Harvey SM (1987). The causes of buoyancy in eggs of marine teleosts. J Mar Biol Assoc U K.

[20] Greeley MS, Calder DR, Wallace RA (1991). Changes in size, hydration and low molecular weight osmotic effectors during meiotic maturation of Fundulus oocytes in vivo. Comp Biochem Physiol.

[21] Thorsen A, Fyhn HJ. Osmotic effectors during preovulatory swelling of marine fish eggs. In: Scott AP, Sumpter JP, Kime DE, Rolfe MS, editors. Proceedings of the fourth international symposium on the reproductive physiology of fish: Sheffield; 1991. p. 312–4.

[22] Matsubara T, Ohkubo N, Andoh T, Sullivan CV, Hara A (1999). Two forms of vitellogenin, yielding two distinct lipovitellins, play different roles during oocyte maturation and early development of barfin flounder, *Verasper moseri*, a marine teleost spawning pelagic eggs. Dev Biol.

[23] Fyhn HJ, Serigstad B (1987). Free amino acids as energy substrate in developing eggs and larvae of the cod *Gadus morhua*. Mar Biol.

[24] Rønnestad I, Fyhn HJ (1993). Metabolic aspects of free amino acids in developing marine fish eggs and larvae. Rev Fish Sci.

[25] Rønnestad I, Groot EP, Fyhn HJ (1993). Compartmental distribution of free amino acids and protein in developing yolk-sac larvae of Atlantic halibut (*Hippoglossus hippoglossus*). Mar Biol.

[26] Rønnestad I, Robertson R, Fyhn HJ. Free amino acids and protein content in pelagic and demersal eggs of tropical marine fishes. In: DD MK, Eldridge M, editors. The Fish Egg: American Fisheries Society; 1996. p. 81–4.

[27] Rønnestad I, Finn RN, Thorsen A. Fish larval nutrition: recent advances in amino acid metabolism. Aquaculture. 1999;177(1-4):201–16. 10.1016/S0044-8486(99)00082-4.

[28] Finn RN, Rønnestad I, Fyhn HJ (1995). Respiration, nitrogen and energy metabolism of developing yolk-sac larvae of Atlantic halibut (Hippoglossus hippoglossus L.). comp. Biochem. Physiol..

[29] Finn RN, Fyhn HJ, Evjen ME. Physiological energetics of developing embryos and yolk-sac larvae of Atlantic cod (*Gadus morhua*). I. Respiration and nitrogen metabolism. Mar. Biol. 1995;124(3):355–69. 10.1007/BF00363909.

[30] Matsubara T, Nagae M, Ohkubo N, Andoh T, Sawaguchi S, Hiramatsu N, et al. Multiple vitellogenins and their unique roles in marine teleosts. Fish Physiol Biochem. 2003;28(1-4):295–9. 10.1023/B:FISH.0000030559.71954.37.

[31] Williams VN, Reading BJ, Hiramatsu N, Amano H, Glassbrook N, Hara A, et al. Multiple vitellogenins and product yolk proteins in striped bass, *Morone saxatilis*: molecular characterization and processing during oocyte growth and maturation. Fish Physiol Biochem. 2014;40(2):395–415. 10.1007/s10695-013-9852-0. 10.1007/s10695-013-9852-0 24005815

[32] Yilmaz O, Prat F, Ibáñez JA, Koksoy S, Amano H, Sullivan CV. Multiple vitellogenins and product yolk proteins in European sea bass (*Dicentrarchus labrax*): Molecular characterization, quantification in plasma, liver and ovary, and maturational proteolysis. Comp Biochem Physiol. 2016;Part B. 194:71–86. 10.1016/j.cbpb.2015.11.010. 10.1016/j.cbpb.2015.11.010 26643259

[33] Yilmaz O, Patinote A, Nguyen T, Com E, Pineau C, Bobe J. Genome editing reveals reproductive and developmental dependencies on specific types of vitellogenin in zebrafish (*Danio rerio*). Mol Reprod Dev. 2019;86(9):1168–88. 10.1002/mrd.23231. 10.1002/mrd.23231 31380595

[34] Yilmaz O, Patinote A, Nguyen T, Com E, Lavigne R, Pineau C, et al. Scrambled eggs: proteomic portraits and novel biomarkers of egg quality in zebrafish (*Danio rerio*). PLoS One. 2017;12(11):e0188084. 10.1371/journal.pone.0188084. 10.1371/journal.pone.0188084 PMC5690628 29145436

[35] Tyler CR. Vitellogenesis in salmonids. In: Scott AP, Sumpter JP, Kime DE, Rolfe J, editors. Proc. Fourth Int. Symp. Reprod. Physiol. of Fish. Sheffield: Sheffield University Press; 1991. p. 295–9.

[36] Link V, Shevchenko A, Heisenberg C-P. Proteomics of early zebrafish embryos. BMC Dev Biol. 2006;6(1):1. 10.1186/1471-213X-6-1. 10.1186/1471-213X-6-1 PMC1363346 16412219

[37] Arribat Y, Grepper D, Lagarrigue S, Richard J, Gachet M, Gut P, et al. Mitochondria in embryogenesis: an Organellogenesis perspective. Front Cell Dev Biol. 2019;7:282. 10.3389/fcell.2019.00282. 10.3389/fcell.2019.00282 PMC6883342 31824944

[38] Buszczak M, Signer RAJ, Morrison SJ. Cellular differences in protein synthesis regulate tissue homeostasis. Cell. 2014;159(2):242–51. 10.1016/j.cell.2014.09.016. 10.1016/j.cell.2014.09.016 PMC4222182 25303523

[39] Lee G, Hynes R, Kirschner M (1984). Temporal and spatial regulation of fibronectin in early Xenopus development. Cell..

[40] Norris ML, Barton SC, Surani MAH (1985). Changes in protein synthesis during early cleavage of the Mongolian gerbil embryo. J Exp Zool Developmental and Cellular Biology.

[41] Crosby IM, Gandolfi F, Moor RM (1988). Control of protein synthesis during early cleavage of sheep embryos. J Reprod Fertil.

[42] Tata JR (1986). Coordinated assembly of the developing egg. BioEssays..

[43] Brooks S, Tyler CR, Sumpter JP (1997). Egg quality in fish: what makes a good egg?. Rev Fish Biol Fish.

[44] Peshkin L, Wühr M, Pearl E, Haas W, Freeman RM Jr, Gerhart JC, et al. On the relationship of protein and mRNA dynamics in vertebrate embryonic development. Dev Cell. 2015;35(3):383–94. 10.1016/j.devcel.2015.10.010. 10.1016/j.devcel.2015.10.010 PMC4776761 26555057

[45] Lin T, Lee JE, Kang JW, Shin HY, Lee JB, Jin DI (2019). Endoplasmic reticulum (ER) stress and unfolded protein response (UPR) in mammalian oocyte maturation and Preimplantation embryo development. Int J Mol Sci.

[46] Terrab L, Wipf P (2020). Hsp70 and the unfolded protein response as a challenging drug target and an inspiration for probe molecule development. ACS Med Chem Lett.

[47] Gardner BM, Pincus D, Gotthardt K, Gallagher CM, Walter P. Endoplasmic reticulum stress sensing in the unfolded protein response. Cold Spring Harb Perspect Biol. 2013;5(3):a013169. 10.1101/cshperspect.a013169. 10.1101/cshperspect.a013169 PMC3578356 23388626

[48] Stacchiotti A (2019). Exploring cellular stress response and chaperones. Cells..

[49] Wilkinson B, Gilbert HF (1699). Protein disulfide isomerase. Biochim Biophys Acta.

[50] Ma H, Martin K, Dixon D, Hernandez AG, Weber GM (2019). Transcriptome analysis of egg viability in rainbow trout, *Oncorhynchus mykiss*. BMC Genomics.

[51] Kim K, Kenigsberg S, Jurisicova A, Bentov Y (2019). The role of mitochondria in oocyte and early embryo health. OBM Genetics.

[52] Wai T, Ao A, Zhang XY, Cyr D, Dufort D, Shoubridge EA (2010). The role of mitochondrial DNA copy number in mammalian fertility. Biol Reprod.

[53] Ge HS, Tollner TL, Hu Z, Dai MM, Li XH, Guan HQ, Shan D, Zhang X, Lv J, Huang C, Dong Q (2012). The importance of mitochondrial metabolic activity and mitochondrial DNA replication during oocyte maturation in vitro on oocyte quality and subsequent embryo developmental competence. Mol Reprod Dev.

[54] Pikó L, Matsumoto L (1976). Number of mitochondria and some properties of mitochondrial DNA in the mouse egg. Dev Biol.

[55] Pikó L, Taylor KD (1987). Amounts of mitochondrial DNA and abundance of some mitochondrial gene transcripts in early mouse embryos. Dev Biol.

[56] Artuso L, Romano A, Verri T, Domenichin A, Argenton F, Santorelli FM, Petruzzella V (2012). Mitochondrial DNA metabolism in early development of zebrafish (*Danio rerio*). BBA-Bioenergetics..

[57] Chappel S (2013). The role of mitochondria from mature oocyte to viable blastocyst. Obstet Gynecol Int.

[58] Dumollard R, Duchen M, Sardet C (2006). Calcium signals and mitochondria at fertilisation. Semin Cell Dev Biol.

[59] Wendling NC, Bencic DC, Nagler JJ, Cloud JG, Ingermann RL (2004). Adenosine triphosphate levels in steelhead (Oncorhynchus mykiss) eggs: an examination of turnover, localization and role. Comp Biochem Phys A.

[60] Dutta A, Sinha DK (2017). Zebrafish lipid droplets regulate embryonic ATP homeostasis to power early development. Open Biol.

[61] Van der Plas-Duivesteijn S, Mohammed Y, Dalebout H, Meijer A, Botermans A, Hoogendijk JL, et al. Identifying proteins in Zebrafish embryos using spectral libraries generated from dissected adult organs and tissues. J Proteome Res. 2014;13(3):1537–44. 10.1021/pr4010585. 10.1021/pr4010585 24460240

[62] Lahnsteiner F, Soares F, Ribeiro L, Dinis MT. Egg quality determination in teleost fish. In: Cabrita E, Herraez P, Robles V, editors. Methods in Reproductive Aquaculture, Marine and Freshwater Species: CRC Press; 2009. p. 149–73.

[63] Lößner C, Wee S, Ler SG, Li RHX, Carney T, Blackstock W, et al. Expanding the zebrafish embryo proteome using multiple fractionation approaches and tandem mass spectrometry. Proteomics. 2012;12(11):1879–82. 10.1002/pmic.201100576. 10.1002/pmic.201100576 22653788

[64] Verri T, Terova G, Romano A, Barca A, Pisani P, Storelli C, Saroglia M. The SoLute carrier (SLC) family series in teleost fish. In: Saroglia M, Liu Z, editors, Functional Genomics in Aquaculture, 2012. doi:10.1002/9781118350041.ch10, The SoLute Carrier (SLC) Family Series in Teleost Fish.

[65] Guo C, Sun L, Chen X, Zhang D (2013). Oxidative stress, mitochondrial damage and neurodegenerative diseases. Neural Regen Res.

[66] Komiya Y, Habas R (2008). Wnt signal transduction pathways. Organogenesis.

[67] Tublin JM, Adelstein JM, del Monte F, Combs CK, Wold LE. Getting to the heart of Alzheimer disease. Circ Res. 2019;124(1):142–9. 10.1161/CIRCRESAHA.118.313563. 10.1161/CIRCRESAHA.118.313563 PMC6319653 30605407

[68] Chen H, Chan DC. Physiological functions of mitochondrial fusion. Ann N Y Acad Sci. 2010;1201(1):21–5. 10.1111/j.1749-6632.2010.05615.x. 10.1111/j.1749-6632.2010.05615.x 20649534

[69] Youle RJ, van der Bliek AM (2012). Mitochondrial fission, fusion, and stress. Science..

[70] Li Q, Velde CV, Israelson A, Xie J, Bailey AO, Dong M-Q, et al. ALS-linked mutant superoxide dismutase 1 (SOD1) alters mitochondrial protein composition and decreases protein import. Proc Natl Acad Sci U S A. 2010;107(49):21146–51. 10.1073/pnas.1014862107. 10.1073/pnas.1014862107 PMC3000256 21078990

[71] Rugarli EI, Langer T (2012). Mitochondrial quality control: a matter of life and death for neurons. EMBO J.

[72] Labbadia J, Morimoto RI (2013). Huntington’s disease: underlying molecular mechanisms and emerging concepts. Trends Biochem Sci.

[73] Williams AJ, Paulson HL (2008). Polyglutamine neurodegeneration: protein misfolding revisited. Trends Neurosci.

[74] Elmore S (2007). Apoptosis: a review of programmed cell death. Toxicol Pathol.

[75] Dong CH, Yang ST, Yang ZA, Zhang L, Gui JF (2004). A C-type lectin associated and translocated with cortical granules during oocyte maturation and egg fertilization in fish. Dev Biol.

[76] Lavigne R, Becker E, Liu Y (2012). Evrard, Lardenois A, Primig M, Pineau C. Direct iterative protein profiling (DIPP)-an innovative method for large-scale protein detection applied to budding yeast mitosis. Mol Cell Proteomics.

[77] Vizcaíno JA, Deutsch EW, Wang R, Csordas A, Reisinger F, Ríos D, et al. ProteomeXchange provides globally co-ordinated proteomics data submission and dissemination. Nat Biotechnol. 2014;30(3):223–6. 10.1038/nbt.2839. 10.1038/nbt.2839 PMC3986813 24727771

[78] Vizcaíno JA, Csordas A, del-Toro N, Dianes JA, Griss J, Lavidas I (2016). Update of the PRIDE database and related tools. Nucleic Acids Res.

[79] Huang DW, Sherman BT, Lempicki RA (2009). Systematic and integrative analysis of large gene lists using DAVID bioinformatics resources. Nat Protoc.

[80] Huang DW, Sherman BT, Lempicki RA (2009). Bioinformatics enrichment tools: paths toward the comprehensive functional analysis of large gene lists. Nucleic Acids Res.

[81] Kanehisa M, Goto S (2000). KEGG: Kyoto encyclopedia of genes and genomes. Nucleic Acids Res.

[82] Kanehisa M (2019). Toward understanding the origin and evolution of cellular organisms. Protein Sci.

[83] Mi H, Poudel S, Muruganujan A, Casagrande JT, Thomas PD (2016). PANTHER version 10: expanded protein families and functions, and analysis tools. Nucleic Acids Res.

[84] Huaiyu M, Thomas P. PANTHER pathway: an ontology-based pathway database coupled with data analysis tools. In: Nikolsky Y, Bryant J, editors. Methods Mol Biol 2009; 563: 123–140. doi: 10.1007/978-1-60761-175-2_7. 10.1007/978-1-60761-175-2_7 PMC6608593 19597783

[85] Szklarczyk D, Franceschini A, Wyder S, Forslund K, Heller D, Huerta-Cepas J, et al. STRING v10: protein-protein interaction networks, integrated over the tree of life. Nucleic Acids Res. 2015;43(D1):D447–52. 10.1093/nar/gku1003. 10.1093/nar/gku1003 PMC4383874 25352553

