# Supplementary material for: Knock out of specific maternal vitellogenins in zebrafish (Danio rerio) evokes vital changes in egg proteomic profiles that resemble the phenotype of poor quality eggs
Source: BMC Genomics. 2021 Apr 28;22:308. doi: 10.1186/s12864-021-07606-1 (PMC8082894; doi:10.1186/s12864-021-07606-1)
Supplement: Supplementary file 1 — Additional file 1. [file 12864_2021_7606_MOESM1_ESM.pdf]

Knock out of specific maternal vitellogenins in zebrafish (*Danio rerio*) evokes vital changes in egg proteomic profiles that resemble the phenotype of poor quality eggs

Ozlem Yilmaz[12*], Amelie Patinote[1], Emmanuelle Com[34], Charles Pineau[34], Julien Bobe[1]

[1] INRAE, LPGP, 35000, Rennes, France.

[2]Institute of Marine Research, Austevoll Research Station, Storebø, Norway

[3]Univ Rennes, Inserm, EHESP, Irset-UMR_S 1085, F-35042 Rennes cedex, France

[4]Protim, Univ Rennes, F-35042 Rennes cedex, France

* Correspondence: ozlem.yilmaz@hi.no

Table S1. Proteins differentially regulated in the *vtg1*-KO experiment. List of the 126 proteins from the *vtg1*-KO experiment that were considered to be differentially regulated between wild type (Wt) and *vtg1*-KO eggs and whose distribution among various functional categories is illustrated in Fig 1a. These include proteins detected only in wild type eggs (unique in Wt, N=2), proteins detected in Wt eggs with N-SC>1.5-fold relative to values for *vtg1*-KO eggs (down-regulated in KO, N=30), proteins detected only in *vtg1*-KO eggs (unique in KO, N=19), and proteins detected in *vtg1*-KO eggs with N-SC>1.5-fold relative to values for Wt eggs (up-regulated in KO, N=75). For each protein, the Ensembl Protein ID and associated gene, transcript and protein name, functional category, Regulation (compared to Wt) (unique, up-regulated in KO, or down-regulated in KO), and fold-difference in N-SC between groups (if available) is shown. Color shading corresponds to that used to designate functional categories in Fig 1.

	Ensembl Protein ID	Associated Gene Name	Associated Transcript Name	Protein full name	Functional Category	Regulation (compared to Wt)	Fold Difference
1	ENSDARP00000101309	vtg4	vtg4-201	Vitelogenin 4	Vitellogenins	unique in Wt	-
2	ENSDARP00000115012	vtg4	vtg4-204	Vitelogenin 4	Vitellogenins	unique in Wt	-
3	ENSDARP00000072738	vtg1	vtg1-201	Vitellogenin 1	Vitellogenins	down-regulated in KO	1427.37
4	ENSDARP00000050237	vtg1	vtg1-001	Vitellogenin 1	Vitellogenins	down-regulated in KO	1150.36
5	ENSDARP00000072687	vtg5	vtg5-201	Vitellogenin 5	Vitellogenins	down-regulated in KO	26.51
6	ENSDARP00000109480	vtg5	vtg5-202	Vitellogenin 5	Vitellogenins	down-regulated in KO	18.55
7	ENSDARP00000013402	cat	cat-201	Catalase	Redox/Detox related	down-regulated in KO	4.53
8	ENSDARP00000107673	cat	cat-202	Catalase	Redox/Detox related	down-regulated in KO	4.53
9	ENSDARP00000040396	prdx2	prdx2-201	Peroxiredoxin 2	Redox/Detox related	down-regulated in KO	3.57
10	ENSDARP00000124779	cat	cat-001	Catalase	Redox/Detox related	down-regulated in KO	3.13
11	ENSDARP00000126962	ckbb	ckbb-203	Creatine kinase, brain b	Energy metabolism	down-regulated in KO	2.83
12	ENSDARP00000115226	ckbb	ckbb-204	Creatine kinase, brain b	Energy metabolism	down-regulated in KO	2.83
13	ENSDARP00000126677	ckbb	ckbb-205	Creatine kinase, brain b	Energy metabolism	down-regulated in KO	2.64
14	ENSDARP00000113177	crp	crp-201	C-reactive protein	Immune system related	down-regulated in KO	2.48
15	ENSDARP00000073460	crp2	crp2-203	C-reactive protein 2	Immune system related	down-regulated in KO	2.48
16	ENSDARP00000128605	slc45a4	slc45a4-203	Solute carrier family 45, member 4	Energy metabolism	down-regulated in KO	2.28
17	ENSDARP00000080196	slc45a4	slc45a4-201	Solute carrier family 45, member 4	Energy metabolism	down-regulated in KO	2.27
18	ENSDARP00000097762	si:dkey-46g23.2	si:dkey-46g23.2-202	Alpha-2-macroglobulin-like 1	Protein degradation and synthesis inhibition	down-regulated in KO	2.26
19	ENSDARP00000024900	ckbb	ckbb-201	Creatine kinase, brain b	Energy metabolism	down-regulated in KO	1.90
20	ENSDARP00000026065	hsp90aa1.2	hsp90aa1.2-201	Heat shock protein 90, alpha (cytosolic), class A member 1, tandem duplicate 2	Protein degradation and synthesis inhibition	down-regulated in KO	1.82
21	ENSDARP00000116170	zgc:113984	zgc:113984-202	SUEL type lectin	Lectins	down-regulated in KO	1.73
22	ENSDARP00000121130	zgc:113984	zgc:113984-203	SUEL type lectin	Lectins	down-regulated in KO	1.73
23	ENSDARP00000122705	zgc:113984	zgc:113984-204	SUEL type lectin	Lectins	down-regulated in KO	1.72
24	ENSDARP00000005260	MFAP4 (8 of 14)	MFAP4 (8 of 14)-201	Microfibrillar-associated protein 4	Cell cycle, division, growth and fate	down-regulated in KO	1.69
25	ENSDARP00000126800	ckbb	ckbb-207	Creatine kinase, brain b	Energy metabolism	down-regulated in KO	1.65
26	ENSDARP00000015917	ckba	ckba-201	Creatine kinase, brain a	Energy metabolism	down-regulated in KO	1.61
27	ENSDARP00000092517	ckba	ckba-202	Creatine kinase, brain a	Energy metabolism	down-regulated in KO	1.61
28	ENSDARP00000124582	cnp	cnp-011	2,,3,-cyclic nucleotide 3, phosphodiesterase	Cell cycle, division, growth and fate	down-regulated in KO	1.58
29	ENSDARP00000124216	cnp	cnp-007	2,,3,-cyclic nucleotide 3, phosphodiesterase	Cell cycle, division, growth and fate	down-regulated in KO	1.58
30	ENSDARP00000056380	crp2	crp2-201	C-reactive protein 2	Immune reponse related	down-regulated in KO	1.55
31	ENSDARP00000114321	si:dkey-90l23.2	si:dkey-90l23.2-203	SUEL type lectin	Lectins	down-regulated in KO	1.52
32	ENSDARP00000116297	si:dkey-90l23.2	si:dkey-90l23.2-202	SUEL type lectin	Lectins	down-regulated in KO	1.52
33	ENSDARP00000063799	gapdh	gapdh-201	Glyceraldehyde-3-phosphate dehydrogenase	Energy metabolism	up-regulated in KO	0.65
34	ENSDARP00000023158	vtg3	vtg3-201	Vitelogenin 3	Vitellogenins	up-regulated in KO	0.62
35	ENSDARP00000013329	ldhba	ldhba-201	Lactate dehydrogenase Ba	Energy metabolism	up-regulated in KO	0.62
36	ENSDARP00000098462	zgc:165518	zgc:165518-201	Alpha-2-macroglobulin-like 1	Protein degradation and synthesis inhibition	up-regulated in KO	0.62
37	ENSDARP00000011595	gyg1a	gyg1a-201	Glycogenin 1a	Energy metabolism	up-regulated in KO	0.62
38	ENSDARP00000102385	zgc:165518	zgc:165518-202	Alpha-2-macroglobulin-like 1	Protein degradation and synthesis inhibition	up-regulated in KO	0.62
39	ENSDARP00000094194	vtg3	vtg3-202	Vitellogenin 3, phosvitinless	Vitellogenins	up-regulated in KO	0.60
40	ENSDARP00000119867	hspa8	hspa8-202	Heat shock protein 8	Protein degradation and synthesis inhibition	up-regulated in KO	0.60
41	ENSDARP00000015293	cp	cp-201	Ceruloplasmin	Redox/Detox related	up-regulated in KO	0.59
42	ENSDARP00000010413	hsp70.3	hsp70.3-201	Heat shock cognate 70-kd protein, tandem duplicate 3	Protein degradation and synthesis inhibition	up-regulated in KO	0.58
43	ENSDARP00000030050	hsp70l	hsp70l-201	Heat shock cognate 70-kd protein, like	Protein degradation and synthesis inhibition	up-regulated in KO	0.58
44	ENSDARP00000045766	mcm5	mcm5-201	MCM5 minichromosome maintenance deficient 5	Cell cycle, division, growth and fate	up-regulated in KO	0.58
45	ENSDARP00000103854	hsp70.2	hsp70.2-202	Heat shock cognate 70-kd protein, tandem duplicate 2	Protein degradation and synthesis inhibition	up-regulated in KO	0.58
46	ENSDARP00000109199	FP016243.1	FP016243.1-201	MCM5 minichromosome maintenance deficient 5	Cell cycle, division, growth and fate	up-regulated in KO	0.58
47	ENSDARP00000128406	vtg3	vtg3-001	Vitellogenin 3, phosvitinless	Vitellogenins	up-regulated in KO	0.56
48	ENSDARP00000049792	gstm.1	gstm.1-201	Glutathione S-transferase M	Redox/Detox related	up-regulated in KO	0.55
49	ENSDARP00000069198	nots	nots-201	Nothepsin	Protein degradation and synthesis inhibition	up-regulated in KO	0.53
50	ENSDARP00000127005	HSPA8 (1 of many)	HSPA8 (1 of many)-203	Heat shock 70kDa protein 8	Protein degradation and synthesis inhibition	up-regulated in KO	0.49
51	ENSDARP00000128683	ACTG2 (1 of many)	ACTG2 (1 of many)-201	Actin, alpha, cardiac muscle 1	Cell cycle, division, growth and fate	up-regulated in KO	0.48
52	ENSDARP00000117697	nasp	nasp-205	Nuclear autoantigenic sperm protein (histone-binding)	Protein synthesis	up-regulated in KO	0.47
53	ENSDARP00000074169	nme2b.2	nme2b.2-201	NME/NM23 nucleoside diphosphate kinase 2b, tandem duplicate 2	Cell cycle, division, growth and fate	up-regulated in KO	0.46
54	ENSDARP00000066429	acta2	acta2-201	Actin, alpha 2, smooth muscle, aorta	Cell cycle, division, growth and fate	up-regulated in KO	0.45
55	ENSDARP00000100195	ACTG2 (1 of many)	ACTG2 (1 of many)-202	Actin, alpha, cardiac muscle 1	Cell cycle, division, growth and fate	up-regulated in KO	0.45
56	ENSDARP00000124371	acta2	acta2-202	Actin, alpha 2, smooth muscle, aorta	Cell cycle, division, growth and fate	up-regulated in KO	0.43
57	ENSDARP00000052837	acta1a	acta1a-201	Actin, alpha 1a, skeletal muscle	Cell cycle, division, growth and fate	up-regulated in KO	0.43
58	ENSDARP00000055135	actc1b	actc1b-201	Actin, alpha, cardiac muscle 1b	Cell cycle, division, growth and fate	up-regulated in KO	0.43
59	ENSDARP00000058628	acta1b	acta1b-201	Actin, alpha, cardiac muscle 1b	Cell cycle, division, growth and fate	up-regulated in KO	0.43
60	ENSDARP00000062369	actc1a	actc1a-201	Actin, alpha 1a, skeletal muscle	Cell cycle, division, growth and fate	up-regulated in KO	0.43
61	ENSDARP00000075110	ACTC1 (1 of many)	ACTC1 (1 of many)-201	Novel actin protein	Cell cycle, division, growth and fate	up-regulated in KO	0.43
62	ENSDARP00000100434	ACTC1 (1 of many)	ACTC1 (1 of many)-202	Novel actin protein	Cell cycle, division, growth and fate	up-regulated in KO	0.43
63	ENSDARP00000002844	ahcy	ahcy-201	S-adenosylhomocysteine hydrolase	Protein degradation and synthesis inhibition	up-regulated in KO	0.42
64	ENSDARP00000060309	atp5b	atp5b-201	ATP synthase, H+ transporting, mitochondrial F1 complex, beta polypeptide	Energy metabolism	up-regulated in KO	0.42
65	ENSDARP00000099319	nme2b.2	nme2b.2-204	NME/NM23 nucleoside diphosphate kinase 2b, tandem duplicate 2	Cell cycle, division, growth and fate	up-regulated in KO	0.41
66	ENSDARP00000118517	gapdh	gapdh-203	Glyceraldehyde-3-phosphate dehydrogenase	Energy metabolism	up-regulated in KO	0.41
67	ENSDARP00000118100	gapdh	gapdh-204	Glyceraldehyde-3-phosphate dehydrogenase	Energy metabolism	up-regulated in KO	0.41
68	ENSDARP00000057284	nasp	nasp-202	Nuclear autoantigenic sperm protein (histone-binding)	Protein synthesis	up-regulated in KO	0.40
69	ENSDARP00000116010	vtg7	vtg7-002	Vitellogenin 7	Vitellogenins	up-regulated in KO	0.40
70	ENSDARP00000089879	ubc	ubc-001	Ubiquitin C	Protein degradation and synthesis inhibition	up-regulated in KO	0.37
71	ENSDARP00000030819	si:ch211-202a12.4	si:ch211-202a12.4-201	Ubiquitin B	Protein degradation and synthesis inhibition	up-regulated in KO	0.37
72	ENSDARP00000106956	si:ch211-202a12.4	si:ch211-202a12.4-202	Ubiquitin B	Protein degradation and synthesis inhibition	up-regulated in KO	0.37
73	ENSDARP00000060744	uba52	uba52-201	Ubiquitin A-52 residue ribosomal protein fusion product 1	Protein degradation and synthesis inhibition	up-regulated in KO	0.37
74	ENSDARP00000088354	ubb	ubb-201	Ubiquitin B	Protein degradation and synthesis inhibition	up-regulated in KO	0.37
75	ENSDARP00000107110	rps27a	rps27a-201	Ribosomal protein S27a	Protein synthesis	up-regulated in KO	0.37
76	ENSDARP00000128775	rps27a	rps27a-204	Ribosomal protein S27a	Protein synthesis	up-regulated in KO	0.37
77	ENSDARP00000072678	vtg7	vtg7-201	Vitellogenin 7	Vitellogenins	up-regulated in KO	0.34
78	ENSDARP00000114558	vtg7	vtg7-001	Vitellogenin 7	Vitellogenins	up-regulated in KO	0.34
79	ENSDARP00000098065	nme2b.2	nme2b.2-203	NME/NM23 nucleoside diphosphate kinase 2b, tandem duplicate 2	Cell cycle, division, growth and fate	up-regulated in KO	0.33
80	ENSDARP00000101082	nme2b.2	nme2b.2-202	NME/NM23 nucleoside diphosphate kinase 2b, tandem duplicate 2	Cell cycle, division, growth and fate	up-regulated in KO	0.33
81	ENSDARP00000104702	qdprb2	qdprb2-203	Quinoid dihydropteridine reductase b2	Redox/Detox related	up-regulated in KO	0.33
82	ENSDARP00000093898	p4hb	p4hb-001	Protein disulfide-isomerase	Protein degradation and synthesis inhibition	up-regulated in KO	0.32
83	ENSDARP00000103251	qdprb2	qdprb2-201	Quinoid dihydropteridine reductase b2	Redox/Detox related	up-regulated in KO	0.31
84	ENSDARP00000063661	mdh2	mdh2-201	Malate dehydrogenase 2, NAD (mitochondrial)	Energy metabolism	up-regulated in KO	0.28
85	ENSDARP00000002751	eef1a1a	eef1a1a-201	Eukaryotic translation elongation factor 1 alpha 1a	Protein synthesis	up-regulated in KO	0.27
86	ENSDARP00000117259	MFAP4 (12 of 14)	MFAP4 (12 of 14)-201	Microfibrillar-associated protein 4	Cell cycle, division, growth and fate	up-regulated in KO	0.24
87	ENSDARP00000124591	pdia3	pdia3-002	Protein disulfide isomerase family A, member 3	Protein degradation and synthesis inhibition	up-regulated in KO	0.22
88	ENSDARP00000125214	si:dkeyp-98a7.8	si:dkeyp-98a7.8-201	SUEL type lectin	Lectins	up-regulated in KO	0.21
89	ENSDARP00000110519	AL929192.1	AL929192.1-201	uncharacterized	Others	up-regulated in KO	0.21
90	ENSDARP00000073345	si:dkey-46g23.5	si:dkey-46g23.5-202	Alpha-2-macroglobulin-like 1	Protein degradation and synthesis inhibition	up-regulated in KO	0.21
91	ENSDARP00000008576	si:dkey-46g23.5	si:dkey-46g23.5-201	Alpha-2-macroglobulin-like 1	Protein degradation and synthesis inhibition	up-regulated in KO	0.20
92	ENSDARP00000107611	qdprb2	qdprb2-202	Quinoid dihydropteridine reductase b2	Redox/Detox related	up-regulated in KO	0.18
93	ENSDARP00000112476	qdprb2	qdprb2-201	Quinoid dihydropteridine reductase b2	Redox/Detox related	up-regulated in KO	0.18
94	ENSDARP00000024082	atic	atic-201	5-aminoimidazole-4-carboxamide ribonucleotide formyltransferase/IMP cyclohydı	Cell cycle, division, growth and fate	up-regulated in KO	0.15
95	ENSDARP00000125193	BX649337.1	BX649337.1-201	SUEL type lectin	Lectins	up-regulated in KO	0.15
96	ENSDARP00000125238	si:dkeyp-98a7.3	si:dkeyp-98a7.3-202	SUEL type lectin	Lectins	up-regulated in KO	0.15
97	ENSDARP00000125244	si:dkeyp-98a7.3	si:dkeyp-98a7.3-201	SUEL type lectin	Lectins	up-regulated in KO	0.15
98	ENSDARP00000125496	si:ch211-250e5.9	si:ch211-250e5.9-201	SUEL type lectin	Lectins	up-regulated in KO	0.15
99	ENSDARP00000120702	si:dkeyp-98a7.4	si:dkeyp-98a7.4-202	SUEL type lectin	Lectins	up-regulated in KO	0.15
100	ENSDARP00000106988	si:dkeyp-98a7.4	si:dkeyp-98a7.4-201	Rhamnose binding lectin-like precursor	Lectins	up-regulated in KO	0.15
101	ENSDARP00000107578	si:dkeyp-98a7.4	si:dkeyp-98a7.4-201	SUEL type lectin	Lectins	up-regulated in KO	0.15
102	ENSDARP00000109226	AL929192.2	AL929192.2-201	Uncharacterized	Others	up-regulated in KO	0.15
103	ENSDARP00000111434	si:dkeyp-98a7.5	si:dkeyp-98a7.5-201	SUEL type lectin	Lectins	up-regulated in KO	0.15
104	ENSDARP00000111784	si:dkeyp-98a7.3	si:dkeyp-98a7.3-201	SUEL type lectin	Lectins	up-regulated in KO	0.15
105	ENSDARP00000069823	eef1a1b	eef1a1b-201	Eukaryotic translation elongation factor 1 alpha 1b	Protein synthesis	up-regulated in KO	0.13
106	ENSDARP00000104468	eef1a1a	eef1a1a-202	Eukaryotic translation elongation factor 1 alpha 1a	Protein synthesis	up-regulated in KO	0.13
107	ENSDARP00000118774	eef1a1b	eef1a1b-202	Eukaryotic translation elongation factor 1 alpha 1b	Protein synthesis	up-regulated in KO	0.13
108	ENSDARP00000043687	si:dkey-241l7.2	si:dkey-241l7.2-001	C-type lectin	Immune response related	unique in KO	-
109	ENSDARP00000107173	si:dkey-241l7.5	si:dkey-241l7.5-201	C-type lectin	Lectins	unique in KO	-
110	ENSDARP00000109765	si:dkey-241l7.4	si:dkey-241l7.4-201	C-type lectin	Lectins	unique in KO	-
111	ENSDARP00000127204	si:dkey-241l7.2	si:dkey-241l7.2-002	C-type lectin	Lectins	unique in KO	-
112	ENSDARP00000127261	si:dkey-241l7.4	si:dkey-241l7.4-202	C-type lectin	Lectins	unique in KO	-
113	ENSDARP00000127328	si:dkey-241l7.2	si:dkey-241l7.2-003	C-type lectin	Lectins	unique in KO	-
114	ENSDARP00000127453	si:dkey-241l7.5	si:dkey-241l7.5-202	C-type lectin	Lectins	unique in KO	-
115	ENSDARP00000010921	eef1a2	eef1a2-201	Eukaryotic translation elongation factor 1 alpha 2	Protein synthesis	unique in KO	-
116	ENSDARP00000093318	QDPR (1 of many)	QDPR (1 of many)-201	Quinoid dihydropteridine reductase	Redox/Detox related	unique in KO	-
117	ENSDARP00000103358	serpina1	serpina1-201	Serine (or cysteine) proteinase inhibitor, clade A (alpha-1 antiproteinase, antitry	Protein degradation and synthesis inhibition	unique in KO	-
118	ENSDARP00000104927	crp2	crp2-204	C-reactive protein 2	Immune response related	unique in KO	-
119	ENSDARP00000107615	serpina1l	serpina1l-001	Serine (or cysteine) proteinase inhibitor, clade A (alpha-1 antiproteinase, antitry	Protein degradation and synthesis inhibition	unique in KO	-
120	ENSDARP00000115568	si:dkeyp-46h3.8	si:dkeyp-46h3.8-201	SUEL type lectin	Lectins	unique in KO	-
121	ENSDARP00000116589	crp3	crp3-201	C-reactive protein 3	Immune response related	unique in KO	-
122	ENSDARP00000122542	ddx41	ddx41-201	DEAD (Asp-Glu-Ala-Asp) box polypeptide 41	Immune response related	unique in KO	-
123	ENSDARP00000122730	ddx41	ddx41-202	DEAD (Asp-Glu-Ala-Asp) box polypeptide 41	Immune response related	unique in KO	-
124	ENSDARP00000123406	si:dkeyp-46h3.8	si:dkeyp-46h3.8-201	SUEL type lectin	Lectins	unique in KO	-
125	ENSDARP00000127700	serpina1l	serpina1l-201	Serine (or cysteine) proteinase inhibitor, clade A (alpha-1 antiproteinase, antitry	Protein degradation and synthesis inhibition	unique in KO	-
126	ENSDARP00000128898	serpina1	serpina1-202	Serine (or cysteine) proteinase inhibitor, clade A (alpha-1 antiproteinase, antitry	Protein degradation and synthesis inhibition	unique in KO	-

Table S2. Proteins differentially regulated in the *vtg3*-KO experiment. List of the 74 proteins from the *vtg3*-KO experiment that were considered to be differentially regulated between wild type (Wt) and *vtg3*-KO eggs and whose distribution among various functional categories is illustrated in Fig 1b. These include proteins detected only in wild type eggs (unique in Wt, N=9), proteins detected in Wt eggs with N-SC >1.5-fold relative to values for *vtg3*-KO eggs (down-regulated in KO, N=12), proteins detected only in *vtg3*-KO eggs (unique in KO, N=1), and proteins detected in *vtg3*-KO eggs with N-SC >1.5-fold relative to values for Wt eggs (up-regulated in KO, N=52). For each protein, the Ensembl Protein ID and associated gene, transcript and protein name, functional category, Regulation (compared to Wt)(unique, up-regulated in KO, or down-regulated in KO), and fold-difference in N-SC between groups (if available) is shown. Color shading corresponds to that used to designate functional categories in Fig 1.

	Ensembl Protein ID	Associated Gene Name	Associated Transcript Name	Protein Full Name	Functional Category	Regulation (compared to Wt)	Fold Difference
1	ENSDARP00000023158	vtg3	vtg3-201	Vitellogenin 3	Vitellogenins	unique in Wt	-
2	ENSDARP00000080196	slc45a4	slc45a4-001	Solute carrier family 45, member 4	Energy metabolism	unique in Wt	-
3	ENSDARP00000089804	zgc:136254	zgc:136254-001	Phosphatidylinositol-3-phosphate binding activity	Lipid metabolism	unique in Wt	-
4	ENSDARP00000089806	si:ch211-251f6.7	si:ch211-251f6.7-201	Phosphatidylinositol-3-phosphate binding activity	Lipid metabolism	unique in Wt	-
5	ENSDARP00000094194	vtg3	vtg3-202	Vitellogenin 3	Vitellogenins	unique in Wt	-
6	ENSDARP00000113985	si:ch211-251f6.7	si:ch211-251f6.7-001	Phosphatidylinositol-3-phosphate binding activity	Lipid metabolism	unique in Wt	-
7	ENSDARP00000128406	vtg3	vtg3-001	Vitellogenin 3, phosvitinless	Vitellogenins	unique in Wt	-
8	ENSDARP00000128605	slc45a4	slc45a4-201	Solute carrier family 45, member 4	Energy metabolism	unique in Wt	-
9	ENSDARP00000129497	zgc:136254	zgc:136254-201	Phosphatidylinositol-3-phosphate binding activity	Lipid metabolism	unique in Wt	-
10	ENSDARP00000013402	cat	cat-201	Catalase	Redox/Detox related	down-regulated in KO	7.26
11	ENSDARP00000107673	cat	cat-202	Catalase	Redox/Detox related	down-regulated in KO	7.26
12	ENSDARP00000124779	cat	cat-001	Catalase	Redox/Detox related	down-regulated in KO	4.97
13	ENSDARP00000115703	eno3	eno3-005	Enolase 3	Energy metabolism	down-regulated in KO	4.27
14	ENSDARP00000029296	eef1a1l1	eef1a1l1-202	Eukaryotic translation elongation factor 1 alpha 1, like 1	Protein synthesis	down-regulated in KO	2.69
15	ENSDARP00000111742	eef1a1l1	eef1a1l1-203	Eukaryotic translation elongation factor 1 alpha 1, like 1	Protein synthesis	down-regulated in KO	2.69
16	ENSDARP00000006339	eef1a1l1	eef1a1l1-201	Eukaryotic translation elongation factor 1 alpha 1, like 1	Protein synthesis	down-regulated in KO	2.44
17	ENSDARP00000120742	eno3	eno3-001	Enolase 3	Energy metabolism	down-regulated in KO	2.39
18	ENSDARP00000017456	hspa5	hspa5-201	Heat shock protein 5	Protein degradation and synthesis inhibition	down-regulated in KO	2.08
19	ENSDARP00000056380	crp2	crp2-001	C-reactive protein 2	Immune response related	down-regulated in KO	1.80
20	ENSDARP00000124216	cnp	cnp-007	2'3'-cyclic nucleotide 3, phosphodiesterase	Cell cycle, division, growth and fate	down-regulated in KO	1.72
21	ENSDARP00000124582	cnp	cnp-011	2'3'-cyclic nucleotide 3, phosphodiesterase	Cell cycle, division, growth and fate	down-regulated in KO	1.72
22	ENSDARP00000072687	vtg5	vtg5-001	Vitellogenin 5	Vitellogenins	up-regulated in KO	0.67
23	ENSDARP00000043742	HSPA8 (1 of many)	HSPA8 (1 of many)-001	Heat shock 70kDa protein 8	Protein degradation and synthesis inhibition	up-regulated in KO	0.65
24	ENSDARP00000114558	vtg7	vtg7-001	Vitellogenin 7	Vitellogenins	up-regulated in KO	0.65
25	ENSDARP00000072678	vtg7	vtg7-201	Vitellogenin 7	Vitellogenins	up-regulated in KO	0.65
26	ENSDARP00000128683	ACTC1 (1 of many)	ACTC1 (1 of many)-001	Actin, alpha, cardiac muscle 1	Cell cycle, division, growth and fate	up-regulated in KO	0.64
27	ENSDARP00000107175	si:ch73-160p18.3	si:ch73-160p18.3-001	Uncharacterized	Others	up-regulated in KO	0.64
28	ENSDARP00000124371	acta2	acta2-001	Actin, alpha 2, smooth muscle, aorta	Cell cycle, division, growth and fate	up-regulated in KO	0.64
29	ENSDARP00000100195	ACTC1 (1 of many)	ACTC1 (1 of many)-002	Actin, alpha, cardiac muscle 1	Cell cycle, division, growth and fate	up-regulated in KO	0.62
30	ENSDARP00000109480	vtg5	vtg5-201	Vitellogenin 5	Vitellogenins	up-regulated in KO	0.61
31	ENSDARP00000052837	acta1a	acta1a-001	Actin, alpha 1a, skeletal muscle	Cell cycle, division, growth and fate	up-regulated in KO	0.60
32	ENSDARP00000055135	actc1b	actc1b-201	Actin, alpha, cardiac muscle 1b	Cell cycle, division, growth and fate	up-regulated in KO	0.60
33	ENSDARP00000058628	acta1b	acta1b-001	Actin, alpha 1b, skeletal muscle	Cell cycle, division, growth and fate	up-regulated in KO	0.60
34	ENSDARP00000062369	actc1a	actc1a-001	Actin, alpha, cardiac muscle 1a	Cell cycle, division, growth and fate	up-regulated in KO	0.60
35	ENSDARP00000066429	acta2	acta2-201	Actin, alpha 2, smooth muscle, aorta	Cell cycle, division, growth and fate	up-regulated in KO	0.60
36	ENSDARP00000075110	zgc:86709	zgc:86709-001	Novel actin protein	Cell cycle, division, growth and fate	up-regulated in KO	0.60
37	ENSDARP00000100434	si:ch73-187m15.4	si:ch73-187m15.4-001	Actin, alpha, cardiac muscle 1a	Cell cycle, division, growth and fate	up-regulated in KO	0.60
38	ENSDARP00000004394	si:ch211-199o1.2	si:ch211-199o1.2-201	protein folding chaperone	Protein degradation and synthesis inhibition	up-regulated in KO	0.60
39	ENSDARP00000073132	hsp70.2	hsp70.2-201	Heat shock cognate 70-kd protein, tandem duplicate 2	Protein degradation and synthesis inhibition	up-regulated in KO	0.59
40	ENSDARP00000102412	hsp70.1	hsp70.1-201	Heat shock cognate 70-kd protein, like	Protein degradation and synthesis inhibition	up-regulated in KO	0.59
41	ENSDARP00000010413	hsp70.3	hsp70.3-001	Heat shock cognate 70-kd protein, tandem duplicate 3	Protein degradation and synthesis inhibition	up-regulated in KO	0.59
42	ENSDARP00000030050	hsp70l	hsp70l-001	Heat shock cognate 70-kd protein, like	Protein degradation and synthesis inhibition	up-regulated in KO	0.59
43	ENSDARP00000045766	mcm/hsp70.1	mcm5-201	MCM5 minichromosome maintenance deficient 5 (S. cerevisiae)	Cell cycle, division, growth and fate	up-regulated in KO	0.59
44	ENSDARP00000103854	hsp70.2	hsp70.2-001	Heat shock cognate 70-kd protein, tandem duplicate 2	Protein degradation and synthesis inhibition	up-regulated in KO	0.59
45	ENSDARP00000109199	mcm/hsp70.1	mcm5-001	MCM5 minichromosome maintenance deficient 5 (S. cerevisiae)	Cell cycle, division, growth and fate	up-regulated in KO	0.59
46	ENSDARP00000114162	si:ch211-199o1.2	si:ch211-199o1.2-001	protein folding chaperone	Protein degradation and synthesis inhibition	up-regulated in KO	0.59
47	ENSDARP00000105057	si:dkeyp-46h3.2	si:dkeyp-46h3.2-001	SUEL type Lectin domain	Lectins	up-regulated in KO	0.56
48	ENSDARP00000113693	si:dkeyp-46h3.3	si:dkeyp-46h3.3-001	SUEL type Lectin domain	Lectins	up-regulated in KO	0.56
49	ENSDARP00000122552	ckba	ckba-004	Creatine kinase, brain a	Energy metabolism	up-regulated in KO	0.56
50	ENSDARP00000059365	ckmb	ckmb-001	Creatine kinase, muscle b	Energy metabolism	up-regulated in KO	0.56
51	ENSDARP00000117584	ckba	ckba-003	Creatine kinase, brain a	Energy metabolism	up-regulated in KO	0.56
52	ENSDARP00000037871	ckma	ckma-001	Creatine kinase, muscle a	Energy metabolism	up-regulated in KO	0.56
53	ENSDARP00000117697	nasp	nasp-001	Nuclear autoantigenic sperm protein (histone-binding)	Protein synthesis	up-regulated in KO	0.56
54	ENSDARP00000119867	hspa8	hspa8-002	Heat shock protein 8	Protein degradation and synthesis inhibition	up-regulated in KO	0.53
55	ENSDARP00000005298	chia.4	chia.4-001	Chitinase, acidic.4	Immune response related	up-regulated in KO	0.47
56	ENSDARP00000013329	ldhba	ldhba-001	Lactate dehydrogenase Ba	Energy metabolism	up-regulated in KO	0.46
57	ENSDARP00000057284	nasp	nasp-004	Nuclear autoantigenic sperm protein (histone-binding)	Protein synthesis	up-regulated in KO	0.44
58	ENSDARP00000002844	ahcy	ahcy-001	S-adenosylhomocysteine hydrolase	Protein degradation and synthesis inhibition	up-regulated in KO	0.44
59	ENSDARP00000030819	si:ch211-202a12.4	si:ch211-202a12.4-001	Ubiquitin B	Protein degradation and synthesis inhibition	up-regulated in KO	0.42
60	ENSDARP00000106956	si:ch211-202a12.4	si:ch211-202a12.4-201	Ubiquitin B	Protein degradation and synthesis inhibition	up-regulated in KO	0.42
61	ENSDARP00000089879	ubc	ubc-001	Ubiquitin C	Protein degradation and synthesis inhibition	up-regulated in KO	0.42
62	ENSDARP00000060744	uba52	uba52-001	Ubiquitin A-52 residue ribosomal protein fusion product 1	Protein degradation and synthesis inhibition	up-regulated in KO	0.42
63	ENSDARP00000088354	ubb	ubb-001	Ubiquitin B	Protein degradation and synthesis inhibition	up-regulated in KO	0.42
64	ENSDARP00000107110	rps27a	rps27a-001	Ribosomal protein S27a	Protein synthesis	up-regulated in KO	0.42
65	ENSDARP00000128775	rps27a	rps27a-002	Ribosomal protein S27a	Protein synthesis	up-regulated in KO	0.42
66	ENSDARP00000107746	chia.4	chia.4-202	Chitinase, acidic.4	Immune response related	up-regulated in KO	0.40
67	ENSDARP00000093898	p4hb	p4hb-001	Protein disulfide-isomerase precursor	Protein degradation and synthesis inhibition	up-regulated in KO	0.36
68	ENSDARP00000059885	ldha	ldha-201	Lactate dehydrogenase A4	Energy metabolism	up-regulated in KO	0.34
69	ENSDARP00000127005	HSPA8 (1 of many)	HSPA8 (1 of many)-003	Heat shock 70kDa protein 8	Protein degradation and synthesis inhibition	up-regulated in KO	0.32
70	ENSDARP00000124591	pdia3	pdia3-002	Protein disulfide isomerase family A, member 3	Protein degradation and synthesis inhibition	up-regulated in KO	0.31
71	ENSDARP00000063661	mdh2	mdh2-201	Malate dehydrogenase 2, NAD (mitochondrial)	Energy metabolism	up-regulated in KO	0.29
72	ENSDARP00000107127	si:dkeyp-46h3.6	si:dkeyp-46h3.6-001	SUEL-type Lectin	Lectins	up-regulated in KO	0.29
73	ENSDARP00000018458	pdia4	pdia4-001	Protein disulfide isomerase associated 4	Protein degradation and synthesis inhibition	up-regulated in KO	0.23
74	ENSDARP00000122652	zgc:66313	zgc:66313-005	Alpha-amylase	Energy metabolism	unique in KO	-

Table S3. Complete PANTHER overrepresentation analysis for differentially regulated proteins in the *vtg1*-KO experiment

***vtg1*-KO Experiment: proteins down-regulated in *vtg1*-KO eggs (Wt; N=32, >4 samples >1.5 fold)**
Analysis Type: PANTHER Overrepresentation Test (Released 20190711)
Annotation Version and Release Date: PANTHER version 14.1 Released 2019-03-12
Reference: Danio rerio
Test Type: Fisher's Exact
Correction: Bonferroni correction for multiple testing **($p < 0.05$)**

PANTHER GO-Slim Biological Process	***#REF***	***#Protein***	***Expected***	***Fold***	***+/-***	***P value***
Response to estradiol	23	3	0.01	> 100	+	1.62E-04
Response to organic cyclic compound	34	3	0.01	> 100	+	4.83E-04
Cellular response to chemical stimulus	245	4	0.1	41.29	+	2.58E-03
Unclassified	12906	5	5.1	0.98	-	0.00E+00

PANTHER GO-Slim Molecular Function	***#REF***	***#Protein***	***expected***	***Fold***	***+/-***	***P value***
Lipid transporter activity	120	3	0.05	63.22	+	5.95E-03
Unclassified	13549	4	5.36	0.75	-	0.00E+00

***vtg1*-KO Experiment: proteins up-regulated in *vtg1*-KO eggs (*vtg1*-KO; N=94, >4 samples >1.5 fold)**
Analysis Type: PANTHER Overrepresentation Test (Released 20190711)
Annotation Version and Release Date: PANTHER version 14.1 Released 2019-03-12
Reference: Danio rerio
Test Type: Fisher's Exact
Correction: Bonferroni correction for multiple testing **($p < 0.05$)**

PANTHER GO-Slim Biological Process	***#REF***	***#Protein***	***Expected***	***Fold***	***+/-***	***P value***
Cellular response to heat	22	3	0.03	98.53	+	0.00766
Response to heat	22	3	0.03	98.53	+	0.00766
Cellular response to unfolded protein	26	3	0.04	83.37	+	0.0121
Response to topologically incorrect protein	41	3	0.06	52.87	+	0.0433
Response to unfolded protein	26	3	0.04	83.37	+	0.0121
Chaperone-mediated protein folding	32	3	0.04	67.74	+	0.0216
Exocytosis	77	5	0.11	46.92	+	0.000133
Vesicle-mediated transport	705	8	0.98	8.2	+	0.00641
Cytokinesis	97	5	0.13	37.24	+	0.000398
Cell division	100	5	0.14	36.13	+	0.000461
Anatomical structure morphogenesis	198	5	0.27	18.25	+	0.0118
Unclassified	12906	8	17.86	0.45	-	0

PANTHER GO-Slim Molecular Function	***#REF***	***#Protein***	***Expected***	***Fold***	***+/-***	***P value***
Heat shock protein binding	28	3	0.04	77.42	+	0.0048
Ubiquitin protein ligase binding	35	3	0.05	61.93	+	0.00894
ATP binding	47	3	0.07	46.12	+	0.0205
Purine ribonucleotide binding	63	3	0.09	34.41	+	0.0472
Nucleotide binding	141	4	0.2	20.5	+	0.0213
Unfolded protein binding	59	3	0.08	36.74	+	0.0392
Structural molecule activity	565	7	0.78	8.95	+	0.00509
Unclassified	13549	6	18.75	0.32	-	0

PANTHER GO-Slim Cellular Component	***#REF***	***#Protein***	***Expected***	***Fold***	***+/-***	***P value***
Actin cytoskeleton	301	5	0.42	12	+	0.0228
Cytosol	724	7	1	6.99	+	0.0204
Unclassified	13836	16	19.15	0.84	-	0

PANTHER Protein Class	***#REF***	***#Protein***	***Expected***	***Fold***	***+/-***	***P value***

Actin and actin related protein	26	5	0.04	> 100	+	0.00000012
Actin family cytoskeletal protein	298	5	0.41	12.12	+	0.0117
Translation elongation factor	38	3	0.05	57.04	+	0.00507
RNA binding protein	628	6	0.87	6.9	+	0.0429
Translation initiation factor	77	3	0.11	28.15	+	0.0377
Dehydrogenase	205	4	0.28	14.1	+	0.0394
Unclassified	14588	13	20.19	0.64	-	0
PANTHER Pathways	***#REF***	***#Protein***	***Expected***	***Fold***	***+/-***	***P value***
Cytoskeletal regulation by Rho GTPase	98	5	0.14	36.86	+	0.0000458
Nicotinic acetylcholine receptor signaling pathway	121	5	0.17	29.86	+	0.000125
Cadherin signaling pathway	142	5	0.2	25.44	+	0.000267
Alzheimer disease-presenilin pathway	148	5	0.2	24.41	+	0.000326
Huntington disease	187	6	0.26	23.18	+	0.0000377
Inflammation mediated by chemokine and cytokine signaling pathway	298	5	0.41	12.12	+	0.00884
Wnt signaling pathway	332	5	0.46	10.88	+	0.0146
Unclassified	22310	23	30.88	0.74	-	0

Table S4. Complete PANTHER Overrepresentation analysis for differentially regulated proteins in the *vtg3*-KO experiment

***vtg3*-KO Experiment: proteins down-regulated in *vtg3*-KO eggs (Wt; N=21 ≥4 samples >1.5 fold)**

Analysis Type: PANTHER Overrepresentation Test (Released 20190711)

Annotation Version and Release Date: PANTHER version 14.1 Released 2019-03-12

Test Type: Fisher's Exact

Reference: Danio rerio

Correction: Bonferroni correction for multiple testing (p<0.05)

PANTHER GO-Slim Biological Process (uncorrected)	***#REF***	***#Protein***	***Expected***	***Fold***	***+/-***	***P value***
Carbohydrate metabolic process	204	1	0.05	20.66	+	4.77E-02
Monosaccharide metabolic process	87	1	0.02	48.45	+	2.07E-02
Response to organic cyclic compound	34	1	0.01	> 100	+	8.27E-03
Response to estradiol	23	1	0.01	> 100	+	5.68E-03

PANTHER GO-Slim Molecular Function	***#REF***	***#Protein***	***Expected***	***Fold***	***+/-***	***P value***
Phosphatidylinositol-3-phosphate binding	13	2	0	> 100	+	2.23E-03
Phosphatidylinositol phosphate binding	40	2	0.01	> 100	+	1.82E-02
Unclassified	13549	1	3.21	0.31	-	0.00E+00

**PANTHER GO-Slim Cellular Component**	***#REF***	***#Protein***	***Expected***	***Fold***	***+/-***	***P value***
Autophagosome	30	2	0.01	> 100	+	8.79E-03
Lysosomal membrane	33	2	0.01	> 100	+	1.05E-02
Unclassified	13836	4	3.28	1.22	+	0.00E+00

***vtg3*-KO Experiment: proteins up-regulated in *vtg3*-KO eggs (*vtg3*-KO; N=53 ≥4 samples >1.5 fold)**

Analysis Type: PANTHER Overrepresentation Test (Released 20190711)

Annotation Version and Release Date: PANTHER version 14.1 Released 2019-03-12

Test Type: Fisher's Exact

Reference: Danio rerio

Correction: Bonferroni correction for multiple testing (p<0.05)

PANTHER GO-Slim Biological Process	***#REF***	***#Protein***	***Expected***	***Fold***	***+/-***	***P value***
Cellular response to heat	22	5	0.03	> 100	+	1.83E-07
Response to heat	22	5	0.03	> 100	+	1.83E-07
Cellular response to unfolded protein	26	5	0.03	> 100	+	3.85E-07
Response to topologically incorrect protein	41	5	0.05	> 100	+	3.07E-06
Response to unfolded protein	26	5	0.03	> 100	+	3.85E-07
Chaperone-mediated protein folding	32	5	0.04	> 100	+	9.82E-07
Protein folding	123	6	0.15	41.12	+	1.18E-05
Exocytosis	77	6	0.09	65.69	+	8.10E-07
Vesicle-mediated transport	705	11	0.84	13.15	+	4.04E-07
Transport	1403	12	1.66	7.21	+	4.21E-05
Establishment of localization	1403	12	1.66	7.21	+	4.21E-05
Localization	2306	13	2.74	4.75	+	1.17E-03
Cytokinesis	97	6	0.12	52.14	+	3.02E-06
Cell division	100	6	0.12	50.58	+	3.59E-06

Modification-dependent protein catabolic process	81	4	0.1	41.63	+	4.27E-03
Anatomical structure morphogenesis	198	7	0.23	29.8	+	5.04E-06
Anatomical structure development	400	7	0.47	14.75	+	5.48E-04
Developmental process	569	7	0.67	10.37	+	5.53E-03
Endocytosis	326	6	0.39	15.51	+	3.11E-03
Unclassified	12906	6	15.31	0.39	-	0.00E+00
PANTHER GO-Slim Molecular Function	***#REF***	***#Protein***	***Expected***	***Fold***	***+/-***	***P value***
Heat shock protein binding	28	5	0.03	> 100	+	1.73E-07
Ubiquitin protein ligase binding	35	4	0.04	96.34	+	5.80E-05
ATP binding	47	5	0.06	89.68	+	1.86E-06
Purine ribonucleotide binding	63	5	0.07	66.9	+	7.37E-06
Ribonucleotide binding	70	5	0.08	60.21	+	1.21E-05
Nucleotide binding	141	5	0.17	29.89	+	3.42E-04
Small molecule binding	300	5	0.36	14.05	+	1.24E-02
Unfolded protein binding	59	5	0.07	71.44	+	5.41E-06
ATPase activity, coupled	244	5	0.29	17.27	+	4.67E-03
ATPase activity	312	5	0.37	13.51	+	1.49E-02
Structural molecule activity	565	9	0.67	13.43	+	6.30E-06
Unclassified	13549	3	16.07	0.19	-	0.00E+00
PANTHER GO-Slim Cellular Component	***#REF***	***#Protein***	***Expected***	***Fold***	***+/-***	***P value***
Actin cytoskeleton	301	6	0.36	16.8	+	5.33E-04
Organelle	4516	16	5.36	2.99	+	4.62E-03
Cytosol	724	8	0.86	9.31	+	5.93E-04
Unclassified	13836	11	16.41	0.67	-	0.00E+00
PANTHER Protein Class	***#REF***	***#Protein***	***Expected***	***Fold***	***+/-***	***P value***
Amino acid kinase	8	3	0.01	> 100	+	5.02E-05
Actin and actin related protein	26	6	0.03	> 100	+	2.94E-10
Actin family cytoskeletal protein	298	6	0.35	16.97	+	2.71E-04
Cytoskeletal protein	620	6	0.74	8.16	+	1.63E-02
Ribosomal protein	146	4	0.17	23.09	+	5.87E-03
Unclassified	14588	13	17.31	0.75	-	0.00E+00
PANTHER Pathways	***#REF***	***#Protein***	***Expected***	***Fold***	***+/-***	***P value***
Cytoskeletal regulation by Rho GTPase	98	6	0.12	51.61	+	3.50E-07
Nicotinic acetylcholine receptor signaling pathway	121	6	0.14	41.8	+	1.17E-06
Parkinson disease	110	5	0.13	38.32	+	3.55E-05
Cadherin signaling pathway	142	6	0.17	35.62	+	2.93E-06
Alzheimer disease-presenilin pathway	148	6	0.18	34.17	+	3.72E-06
Huntington disease	187	6	0.22	27.05	+	1.43E-05
Apoptosis signaling pathway	161	5	0.19	26.18	+	2.19E-04
Inflammation mediated by chemokine and cytokine signaling pathway	298	6	0.35	16.97	+	2.05E-04
Wnt signaling pathway	332	6	0.39	15.23	+	3.78E-04
Unclassified	22310	18	26.47	0.68	-	0.00E+00

Table S5. Complete network enrichment analyses for differentially regulated proteins which were resolved in a STRING subnetwork in the *vtg1*-KO Experiment. **A)** proteins down-regulated in *vtg1*-KO eggs. **B)** proteins up-regulated in *vtg1*-KO eggs. Only statistically significant results are reported (χ^2, $p<0.05$).

A) *vtg1*-KO Experiment: Network stats for proteins down-regulated in *vtg1*-KO eggs (Wt; N=32)

number of nodes:	17
number of edges:	18
average node degree:	2.12
clustering coefficient:	0.64
expected number of edges:	6
PPI enrichment p-value:	2.75E-05
confidence level:	0.15

KEGG Pathways

pathway	description	protein count	FDR
dre00330	Arginine and proline metabolism	2 of 58	0.0074

Reactome Pathways

pathway	description	protein count	FDR
DRE-71288	Creatine metabolism	2 of 10	0.0001

UniProt Keywords

keyword	description	protein count	FDR
KW-0732	Signal	8 of 3209	0.0075

PFAM Protein Domains

domain	description	protein count	FDR
PF09175	Domain of unknown function (DUF1944)	3 of 8	8.71E-07
PF09172	Domain of unknown function (DUF1943)	3 of 12	1.20E-06
PF01347	Lipoprotein amino terminal region	3 of 13	1.20E-06
PF02807	ATP:guanido phosphotransferase, N-terminal domain	2 of 7	8.38E-05
PF00217	ATP:guanido phosphotransferase, C-terminal catalytic do.	2 of 7	8.38E-05
PF13347	MFS/sugar transport protein	2 of 14	0.00019
PF00354	Pentaxin family	2 of 25	0.00046
PF13385	Concanavalin A-like lectin/glucanases superfamily	2 of 33	0.00069
PF02140	Galactose binding lectin domain	2 of 48	0.0012
PF07690	Major Facilitator Superfamily	2 of 154	0.0107

INTERPRO Protein Domains and Features

domain	description	protein count	FDR
IPR037088	Vitellinogen, beta-sheet shell domain superfamily	3 of 8	1.56E-06
IPR015819	Lipid transport protein, beta-sheet shell	3 of 13	1.56E-06
IPR015817	Vitellinogen, open beta-sheet, subdomain 1	3 of 9	1.56E-06
IPR015816	Vitellinogen, beta-sheet N-terminal	3 of 12	1.56E-06
IPR015258	Vitellinogen, beta-sheet shell	3 of 8	1.56E-06
IPR015255	Vitellinogen, open beta-sheet	3 of 12	1.56E-06
IPR011030	Lipovitellin-phosvitin complex, superhelical domain	3 of 13	1.56E-06
IPR001747	Lipid transport protein, N-terminal	3 of 13	1.56E-06
IPR001759	Pentraxin-related	2 of 21	0.00047
IPR000922	D-galactoside/L-rhamnose binding SUEL lectin domain	2 of 38	0.0013
IPR020846	Major facilitator superfamily domain	2 of 148	0.0161
IPR036259	MFS transporter superfamily	2 of 177	0.0208

SMART Protein Domains

domain	description	protein count	FDR
SM01170	Members of this family adopt a structure consisting of several large open beta-sheets. Their exact function has not, as yet, been determined [PUBMED:12135361].	3 of 8	2.90E-07
SM01169	Members of this family adopt a structure consisting of several large open beta-sheets. Their exact function has not, as yet, been determined [PUBMED:12135361].	3 of 12	4.00E-07
SM00638	Lipoprotein N-terminal Domain	3 of 12	4.00E-07

B) *vtg1*-KO Experiment: Network stats for proteins up-regulated in *vtg1*-KO eggs (*vtg1*-KO; N=94)

number of nodes:	63
number of edges:	107
average node degree:	3.4
clustering coefficient:	0.387
expected number of edges:	58
PPI enrichment p-value:	5.95E-09
confidence level:	0.40

KEGG Pathways

pathway	description	protein count	FDR

dre00270	Cysteine and methionine metabolism	3 of 47	0.0072
dre04260	Cardiac muscle contraction	3 of 89	0.0135
dre04141	Protein processing in endoplasmic reticulum	4 of 176	0.0135
dre01100	Metabolic pathways	10 of 1278	0.0135
dre00790	Folate biosynthesis	2 of 26	0.0135
dre00620	Pyruvate metabolism	2 of 41	0.0244
dre03013	RNA transport	3 of 151	0.0263
dre04261	Adrenergic signaling in cardiomyocytes	3 of 180	0.0368
dre00983	Drug metabolism - other enzymes	2 of 61	0.0368
dre00010	Glycolysis / Gluconeogenesis	2 of 74	0.0439

Reactome Pathways

pathway	description	protein count	FDR
DRE-71182	Phenylalanine and tyrosine catabolism	3 of 14	0.003
DRE-450408	AUF1 (hnRNP D0) binds and destabilizes mRNA	4 of 54	0.003
DRE-3371568	Attenuation phase	3 of 19	0.003
DRE-3371497	HSP90 chaperone cycle for steroid hormone receptors	3 of 21	0.003
DRE-6798695	Neutrophil degranulation	7 of 490	0.0088
DRE-168256	Immune System	11 of 1379	0.0154
DRE-3371453	Regulation of HSF1-mediated heat shock response	3 of 69	0.0238
DRE-1430728	Metabolism	12 of 1751	0.0258
DRE-2262752	Cellular responses to stress	5 of 321	0.0313
DRE-168249	Innate Immune System	8 of 875	0.0313
DRE-140837	Intrinsic Pathway of Fibrin Clot Formation	2 of 19	0.0313
DRE-140875	Common Pathway of Fibrin Clot Formation	2 of 23	0.0365
DRE-901042	Calnexin/calreticulin cycle	2 of 25	0.0387

UniProt Keywords

keyword	description	protein count	FDR
KW-0547	Nucleotide-binding	16 of 1588	2.80E-05
KW-0251	Elongation factor	3 of 16	0.00018
KW-0067	ATP-binding	13 of 1287	0.00018
KW-0732	Signal	19 of 3209	0.00099
KW-0520	NAD	4 of 98	0.00099
KW-0676	Redox-active center	2 of 24	0.0095
KW-0560	Oxidoreductase	4 of 349	0.0473

PFAM Protein Domains

domain	description	protein count	FDR
PF02140	Galactose binding lectin domain	13 of 48	7.00E-21
PF00022	Actin	8 of 24	1.82E-13
PF06723	MreB/Mbl protein	4 of 17	3.15E-06
PF00012	Hsp70 protein	4 of 19	3.48E-06
PF03143	Elongation factor Tu C-terminal domain	3 of 9	3.11E-05
PF14560	Ubiquitin-like domain	3 of 10	3.37E-05
PF03144	Elongation factor Tu domain 2	3 of 18	0.00013
PF11976	Ubiquitin-2 like Rad60 SUMO-like	3 of 22	0.0002
PF10569	Alpha-macro-globulin thiol-ester bond-forming region	3 of 22	0.0002
PF07703	Alpha-2-macroglobulin family N-terminal region	3 of 23	0.0002
PF07678	A-macroglobulin complement component	3 of 24	0.0002
PF07677	A-macroglobulin receptor	3 of 24	0.0002
PF01835	MG2 domain	3 of 23	0.0002
PF00207	Alpha-2-macroglobulin family	3 of 23	0.0002
PF00009	Elongation factor Tu GTP binding domain	3 of 35	0.00038
PF00056	lactate/malate dehydrogenase, NAD binding domain	2 of 6	0.00054
PF00240	Ubiquitin family	3 of 42	0.00056
PF02866	lactate/malate dehydrogenase, alpha/beta C-terminal do.	2 of 7	0.00062
PF09175	Domain of unknown function (DUF1944)	2 of 8	0.00073
PF09172	Domain of unknown function (DUF1943)	2 of 12	0.0014
PF01347	Lipoprotein amino terminal region	2 of 13	0.0015
PF13848	Thioredoxin-like domain	2 of 14	0.0017
PF00085	Thioredoxin	2 of 33	0.0077
PF00079	Serpin (serine protease inhibitor)	2 of 33	0.0077
PF00059	Lectin C-type domain	3 of 202	0.0289

INTERPRO Protein Domains and Features

domain	description	protein count	FDR
IPR004001	Actin, conserved site	7 of 12	1.65E-12
IPR020902	Actin/actin-like conserved site	7 of 16	3.99E-12
IPR004000	Actin family	7 of 21	1.27E-11
IPR000922	D-galactoside/L-rhamnose binding SUEL lectin domain	6 of 38	2.57E-08
IPR029048	Heat shock protein 70kD, C-terminal domain superf.	4 of 17	3.01E-06
IPR029047	Heat shock protein 70kD, peptide-binding domain superf.	4 of 16	3.01E-06
IPR018181	Heat shock protein 70, conserved site	4 of 16	3.01E-06
IPR013126	Heat shock protein 70 family	4 of 18	3.01E-06

IPR036291	NAD(P)-binding domain superfamily	7 of 203	8.27E-06
IPR004539	Translation elongation factor EF1A, eukaryotic/archaeal	3 of 5	8.27E-06
IPR019956	Ubiquitin	3 of 9	2.78E-05
IPR019954	Ubiquitin conserved site	3 of 9	2.78E-05
IPR004160	Translation elongation factor EFTu/EF1A, C-terminal	3 of 9	2.78E-05
IPR002353	Type-2 ice-structuring protein	3 of 11	3.60E-05
IPR009001	Translation elongation factor EF1A/initiation factor IF2gamma, C-terminal	3 of 13	5.15E-05
IPR031157	Tr-type G domain, conserved site	3 of 15	7.01E-05
IPR019742	Alpha-2-macroglobulin, conserved site	3 of 17	9.19E-05
IPR004161	Translation elongation factor EFTu-like, domain 2	3 of 18	0.0001
IPR002890	Macroglobulin domain	3 of 19	0.00011
IPR036595	Alpha-macroglobulin, receptor-binding domain superf.	3 of 20	0.00012
IPR011626	Alpha-macroglobulin, TED domain	3 of 20	0.00012
IPR011625	Alpha-2-macroglobulin, bait region domain	3 of 20	0.00012
IPR009048	Alpha-macroglobulin, receptor-binding	3 of 20	0.00012
IPR001599	Alpha-2-macroglobulin	3 of 20	0.00012
IPR008930	Terpenoid cyclases/protein prenyltransferase alpha-alpha toroid	3 of 24	0.00016
IPR000795	Transcription factor, GTP-binding domain	3 of 25	0.00017
IPR009000	Translation protein, beta-barrel domain superfamily	3 of 33	0.00035
IPR001236	Lactate/malate dehydrogenase, N-terminal	2 of 6	0.00061
IPR022383	Lactate/malate dehydrogenase, C-terminal	2 of 7	0.00075
IPR015955	Lactate dehydrogenase/glycoside hydrolase, family 4, Ct	2 of 7	0.00075
IPR001557	L-lactate/malate dehydrogenase	2 of 7	0.00075
IPR037088	Vitellinogen, beta-sheet shell domain superfamily	2 of 8	0.00085
IPR015258	Vitellinogen, beta-sheet shell	2 of 8	0.00085
IPR015817	Vitellinogen, open beta-sheet, subdomain 1	2 of 9	0.00098
IPR011332	Zinc-binding ribosomal protein	2 of 9	0.00098
IPR000626	Ubiquitin domain	3 of 58	0.0013
IPR015816	Vitellinogen, beta-sheet N-terminal	2 of 12	0.0015
IPR015255	Vitellinogen, open beta-sheet	2 of 12	0.0015
IPR015819	Lipid transport protein, beta-sheet shell	2 of 13	0.0016
IPR011030	Lipovitellin-phosvitin complex, superhelical domain	2 of 13	0.0016
IPR001747	Lipid transport protein, N-terminal	2 of 13	0.0016
IPR018378	C-type lectin, conserved site	3 of 81	0.0028
IPR014756	Immunoglobulin E-set	3 of 139	0.0119
IPR001304	C-type lectin-like	3 of 138	0.0119
IPR016186	C-type lectin-like/link domain superfamily	3 of 154	0.0153
IPR016187	C-type lectin fold	3 of 163	0.0174
IPR029071	Ubiquitin-like domain superfamily	3 of 204	0.031
IPR016040	NAD(P)-binding domain	2 of 75	0.0333
IPR005225	Small GTP-binding protein domain	3 of 232	0.0416

SMART Protein Domains

domain	description	protein count	FDR
SM00268	Actin	8 of 24	1.04E-13
SM01361	A-macroglobulin receptor	3 of 24	0.00026
SM01360	Alpha-2-macroglobulin family	3 of 23	0.00026
SM01359	Alpha-2-Macroglobulin	3 of 23	0.00026
SM01170	Members of this family adopt a structure consisting of several large open beta-sheets. Their exact function has not, as yet, been determined [PUBMED:12135361].	2 of 8	0.0007
SM00213	Ubiquitin homologues	3 of 46	0.0007
SM01169	Members of this family adopt a structure consisting of several large open beta-sheets. Their exact function has not, as yet, been determined [PUBMED:12135361].	2 of 12	0.0011
SM00638	Lipoprotein N-terminal Domain	2 of 12	0.0011
SM00093	SERine Proteinase INhibitors	2 of 31	0.005
SM00034	C-type lectin (CTL) or carbohydrate-recognition domain	3 of 202	0.0207

Table S6. Complete network enrichment analyses for differentially regulated proteins which were resolved in a STRING subnetwork in the *vtg3*-KO Experiment. **A)** proteins down-regulated in *vtg3*-KO eggs. **B)** proteins up-regulated in *vtg3*-KO eggs. Only statistically significant results are reported (χ^2, p<0.05).

**A) *vtg3*-KO Experiment: Network stats for proteins down-regulated in *vtg3*-KO eggs (Wt; N=21)**			
number of nodes:	11		
number of edges:	7		
average node degree:	1.27		
clustering coefficient:	0.394		
expected number of edges:	1		
PPI enrichment p-value:	6.99E-06		
confidence level:	0.4		
KEGG Pathways			
**pathway**	**description**	**protein counts**	**FDR**
dre01200	Carbon metabolism	2 of 125	0.0147
PFAM Protein Domains			
**domain**	**description**	**protein counts**	**FDR**
PF06462	Propeller	2 of 7	9.62E-05
PF13347	MFS/sugar transport protein	2 of 14	0.00016
PF07690	Major Facilitator Superfamily	2 of 154	0.0104
INTERPRO Protein Domains and Features			
**domain**	**description**	**protein counts**	**FDR**
IPR006624	Beta-propeller repeat TECPR	2 of 7	0.0002
IPR036259	MFS transporter superfamily	2 of 177	0.0298
IPR020846	Major facilitator superfamily domain	2 of 148	0.0298
SMART Protein Domains			
**domain**	**description**	**protein counts**	**FDR**
SM00706	Beta propeller repeats in Physarum polycephalum tectonins, Limulus lectin L-6 and animal hypothetical proteins.	2 of 8	4.24E-05
**B) *vtg3*-KO Experiment: Network stats for proteins up-regulated in *vtg3*-KO eggs (*vtg3*-KO; N=53)**			
number of nodes:	36		
number of edges:	46		
average node degree:	2.56		
clustering coefficient:	0.417		
expected number of edges:	36		
PPI enrichment p-value:	0.0517		
confidence level:	0.4		
KEGG Pathways			
**pathway**	**description**	**protein count**	**FDR**
dre04141	Protein processing in endoplasmic reticulum	6 of 176	3.04E-06
dre00270	Cysteine and methionine metabolism	4 of 47	6.54E-06
dre00620	Pyruvate metabolism	3 of 41	0.00019
dre00330	Arginine and proline metabolism	3 of 58	0.00037
dre04260	Cardiac muscle contraction	3 of 89	0.001
dre01100	Metabolic pathways	8 of 1278	0.001
dre03040	Spliceosome	3 of 131	0.0022
dre00640	Propanoate metabolism	2 of 31	0.0022
dre04261	Adrenergic signaling in cardiomyocytes	3 of 180	0.0041
dre00010	Glycolysis / Gluconeogenesis	2 of 74	0.0091
dre04144	Endocytosis	3 of 293	0.013
dre04010	MAPK signaling pathway	3 of 359	0.0206
dre03010	Ribosome	2 of 126	0.0206
Reactome Pathways			
**pathway**	**description**	**protein count**	**FDR**
DRE-450408	AUF1 (hnRNP D0) binds and destabilizes mRNA	5 of 54	5.35E-06
DRE-3371568	Attenuation phase	4 of 19	5.35E-06
DRE-3371497	HSP90 chaperone cycle for steroid hormone receptors (SHR)	4 of 21	5.35E-06
DRE-71288	Creatine metabolism	3 of 10	3.53E-05
DRE-3371453	Regulation of HSF1-mediated heat shock response	4 of 69	0.00014
DRE-2262752	Cellular responses to stress	6 of 321	0.0002
DRE-71406	Pyruvate metabolism and Citric Acid (TCA) cycle	3 of 55	0.002
DRE-189085	Digestion of dietary carbohydrate	2 of 11	0.0037
DRE-71291	Metabolism of amino acids and derivatives	4 of 249	0.0076
DRE-901042	Calnexin/calreticulin cycle	2 of 25	0.0111
DRE-70268	Pyruvate metabolism	2 of 31	0.0157
DRE-168256	Immune System	7 of 1379	0.0343

DRE-390522	Striated Muscle Contraction	2 of 54	0.0372
DRE-5358346	Hedgehog ligand biogenesis	2 of 58	0.0408

UniProt Keywords

**keyword**	**description**	**protien count**	**FDR**
KW-0067	ATP-binding	15 of 1287	1.07E-09
KW-0676	Redox-active center	3 of 24	5.07E-05
KW-0520	NAD	4 of 98	6.41E-05
KW-0413	Isomerase	3 of 81	0.00094
KW-0560	Oxidoreductase	3 of 349	0.0462

PFAM Protein Domains

**domain**	**description**	**protien count**	**FDR**
PF00022	Actin	8 of 24	1.62E-15
PF06723	MreB/Mbl protein	5 of 17	1.40E-09
PF00012	Hsp70 protein	5 of 19	1.50E-09
PF00056	lactate/malate dehydrogenase, NAD binding do.	3 of 6	1.50E-06
PF02866	lactate/malate dehydrogenase, alpha/beta C-terminal domain	3 of 7	1.71E-06
PF02807	ATP:guanido phosphotransferase, N-terminal do.	3 of 7	1.71E-06
PF00217	ATP:guanido phosphotransferase, C-terminal catalytic domain	3 of 7	1.71E-06
PF14560	Ubiquitin-like domain	3 of 10	2.54E-06
PF13848	Thioredoxin-like domain	3 of 14	5.35E-06
PF11976	Ubiquitin-2 like Rad60 SUMO-like	3 of 22	1.62E-05
PF00085	Thioredoxin	3 of 33	4.51E-05
PF00240	Ubiquitin family	3 of 42	8.15E-05
PF02140	Galactose binding lectin domain	3 of 48	0.00011
PF01216	Calsequestrin	2 of 7	0.00014
PF09175	Domain of unknown function (DUF1944)	2 of 8	0.00017
PF09172	Domain of unknown function (DUF1943)	2 of 12	0.00031
PF01347	Lipoprotein amino terminal region	2 of 13	0.00034

INTERPRO Protein Domains and Features

**domain**	**description**	**protein count**	**FDR**
IPR004001	Actin, conserved site	7 of 12	1.41E-14
IPR020902	Actin/actin-like conserved site	7 of 16	3.41E-14
IPR004000	Actin family	7 of 21	1.09E-13
IPR029048	Heat shock protein 70kD, C-terminal domain superf.	5 of 17	1.07E-09
IPR029047	Heat shock protein 70kD, peptide-binding domain superf.	5 of 16	1.07E-09
IPR018181	Heat shock protein 70, conserved site	5 of 16	1.07E-09
IPR013126	Heat shock protein 70 family	5 of 18	1.07E-09
IPR001236	Lactate/malate dehydrogenase, N-terminal	3 of 6	1.47E-06
IPR022383	Lactate/malate dehydrogenase, C-terminal	3 of 7	1.87E-06
IPR015955	Lactate dehydrogenase/glycoside hydrolase, family 4, C-terminal	3 of 7	1.87E-06
IPR001557	L-lactate/malate dehydrogenase	3 of 7	1.87E-06
IPR019956	Ubiquitin	3 of 9	2.56E-06
IPR019954	Ubiquitin conserved site	3 of 9	2.56E-06
IPR018177	L-lactate dehydrogenase, active site	2 of 3	7.82E-05
IPR011304	L-lactate dehydrogenase	2 of 3	7.82E-05
IPR000922	D-galactoside/L-rhamnose binding SUEL lectin do.	3 of 38	9.07E-05
IPR005792	Protein disulphide isomerase	2 of 5	0.00014
IPR036802	ATP:guanido phosphotransferase, N-terminal do. Superf.	2 of 6	0.00017
IPR022415	ATP:guanido phosphotransferase active site	2 of 6	0.00017
IPR022414	ATP:guanido phosphotransferase, catalytic domain	2 of 6	0.00017
IPR022413	ATP:guanido phosphotransferase, N-terminal	2 of 6	0.00017
IPR005788	Disulphide isomerase	2 of 7	0.00017
IPR000749	ATP:guanido phosphotransferase	2 of 6	0.00017
IPR037088	Vitellinogen, beta-sheet shell domain superfamily	2 of 8	0.0002
IPR015258	Vitellinogen, beta-sheet shell	2 of 8	0.0002
IPR000626	Ubiquitin domain	3 of 58	0.0002
IPR015817	Vitellinogen, open beta-sheet, subdomain 1	2 of 9	0.00022
IPR011332	Zinc-binding ribosomal protein	2 of 9	0.00022
IPR036291	NAD(P)-binding domain superfamily	4 of 203	0.00034
IPR016040	NAD(P)-binding domain	3 of 75	0.00034
IPR015819	Lipid transport protein, beta-sheet shell	2 of 13	0.00034
IPR015816	Vitellinogen, beta-sheet N-terminal	2 of 12	0.00034
IPR015255	Vitellinogen, open beta-sheet	2 of 12	0.00034
IPR014746	Glutamine synthetase/guanido kinase, catalytic do.	2 of 12	0.00034
IPR011030	Lipovitellin-phosvitin complex, superhelical do.	2 of 13	0.00034
IPR001747	Lipid transport protein, N-terminal	2 of 13	0.00034
IPR017937	Thioredoxin, conserved site	2 of 17	0.0005
IPR013766	Thioredoxin domain	2 of 40	0.0024
IPR029071	Ubiquitin-like domain superfamily	3 of 204	0.0044
IPR017853	Glycoside hydrolase superfamily	2 of 62	0.0052
IPR036249	Thioredoxin-like superfamily	2 of 127	0.0198

SMART Protein Domains

domain	description	protein count	FDR
SM00268	Actin	8 of 24	5.75E-16
SM00213	Ubiquitin homologues	3 of 46	0.00022
SM01170	Members of this family adopt a structure consisting of several large open beta-sheets. Their exact function has not, as yet, been determined [PUBMED:12135361].	2 of 8	0.00029
SM01169	Members of this family adopt a structure consisting of several large open beta-sheets. Their exact function has not, as yet, been determined [PUBMED:12135361].	2 of 12	0.00045
SM00638	Lipoprotein N-terminal Domain	2 of 12	0.00045
